# Synergistic Multi-Source Remote Sensing and Machine Learning for Regional-Scale Soil Salinity Retrieval in the Hyper-Arid Tarim River Basin

**DOI:** 10.3390/s26216851

**Published:** 2026-10-29

**Authors:** Fujun Meng, Aijun Sun, Duojuan Ran, Zhaolin Cao, Shengru Yue

**Affiliations:** 1 College of Water Hydraulic and Architectural Engineering, Tarim University, Alar 843300, China; 120150028@taru.edu.cn (F.M.);; 2 Key Laboratory of Comprehensive Utilization of Saline-Alkali Land, Xinjiang Production and Construction Corps, Alar 843300, China; 3 Tarim Institute of Eco-Environment Protection and Restoration, Alar 843300, China; 4 Natural Resources and Land Control Center, Xinjiang Production and Construction Corps, Urumqi 830000, China

**Keywords:** saline–alkali land, Tarim River Basin, soil salinity mapping, remote sensing, machine learning

## Abstract

**Highlights:**

**What are the main findings?**

- A multi-source RF model achieved R^2^ = 0.73 for soil salinity retrieval in the Tarim River Basin. XGBoost performed better at distinguishing saline from non-saline boundaries at oasis–desert ecotones.
- SHAP analysis showed that climatic variables (MAT and MAP) contributed 33.2% to the model, indicating that climate is a key factor controlling regional salinity patterns.

**What are the implications of the main findings?**

- RF and XGBoost have different strengths. RF is suitable for continuous salinity mapping, while XGBoost works well for boundary extraction. Users can choose based on their actual needs.
- The high-resolution salinity map shows strong spatial heterogeneity (CV = 113.04%) and anthropogenic hotspots in the middle–lower plains, providing baseline data for local land management.

**Abstract:**

Saline–alkali land is known colloquially as the “ringworm of the Earth” because of its wide distribution, difficulty of management, and the serious threat it poses to agricultural production and ecological security in arid regions. The Tarim River Basin is the primary distribution area of saline–alkali land in the arid region of northwest China. Therefore, accurate assessment of its distribution and degree of saline–alkali development is important for the comprehensive management and maintenance of regional ecological security in this region. However, the distribution and characteristics of regional saline–alkali land are unclear because of the limitations of traditional land survey methods. To address this, a field campaign was conducted across the Tarim River Basin, yielding 147 topsoil specimens subsequently subjected to electrical conductivity determinations, and it used a retrieval model for saline–alkali soils by integrating remote sensing techniques and machine learning algorithms. The results show that a Random Forest (RF) model integrating all multi-source features was optimal for soil salinity retrieval, achieving the highest accuracy (R^2^ = 0.73, RMSE = 3.58 dS m^−1^, MAE = 1.89 dS m^−1^), with superior robustness and extreme value prediction, while Extreme Gradient Boosting (XGBoost) complemented saline vs. non-saline boundary extraction. Macro-climatic variables (MAT, MAP) dominated with a 33.2% combined contribution, consistent with the dominant climatic control on soil salinity in hyper-arid regions. Multi-source synergies including optical indices, Sentinel-1 SAR (12.0%), and environmental covariates enhanced physical interpretability. Spatially, soil salinity exhibited strong heterogeneity (CV = 113.04%), being concentrated in the middle–lower plains and irrigation districts affected by anthropogenic activities, contrasting with low-salinity upper mountainous regions. Our results demonstrate that incorporating land survey, remote sensing, and machine learning supports screening-level retrieval of the extent of saline–alkali land in the hyper-arid Tarim River Basin, providing a foundation for its systematic improvement.

## 1. Introduction

Saline–alkali land is defined as containing soil with excessive levels of soluble salts, which adversely affect soil physical, chemical, and biological characteristics, as well as plant growth [1]. It has been metaphorically described as the “ringworm of the Earth” due to its extensive distribution and difficulty of reclamation [2]. Globally, saline–alkali land currently occupies ~1.38 billion hectares, representing roughly 10.7% of Earth’s land surface, with major concentrations in China, the USA, India, Central and Western Asia, and the Mediterranean coastal regions [3,4]. Recently, salinization has intensified considerably, driven by climatic shifts and unsustainable land-use practices [5,6], particularly in hyper-arid regions where it severely compromises soil productivity and ecosystem health. This is especially so in extremely arid regions, where salinization significantly impacts soil productivity and ecological health, causing sustained declines in agricultural output, plant community coverage, and biological diversity [7,8,9]. Approximately one-tenth of global cropland and one-fifth of irrigated systems are presently impacted by salinization, causing yearly economic damages exceeding US$27.3 billion as a result of decreased agricultural productivity [3,10,11]. Therefore, effective management and scientific investigation of saline–alkali land are essential not only for expanding cultivated land resources but also for global ecological security.

In the past two decades, scholarly investigations into salt-affected land have proliferated, using diverse methods [12,13,14]. Conventional approaches to salinity assessment and forecasting relied predominantly on labor-intensive ground-based surveys and specimen collection [15,16]. Li et al. [17] adopted an integrated approach of multi-period electromagnetic induction surveys, layered sampling, laboratory assays, apparent electrical conductivity–target soil (ECa–TS) calibration, and Kriging interpolation to elucidate spatial dynamics of total dissolved salts within a representative arid area of South Xinjiang, China. Yu et al. [18] integrated statistics, geostatistics, and GIS data in the Yellow River Delta to reveal surface salt accumulation and emphasized the critical role of human activities in salt redistribution. However, traditional methods rely on ground sampling and lab-based assays, which, while accurate, are unsuitable for large-scale dynamic monitoring [13,19]. In recent years, remote sensing technology has provided a powerful tool for the spatially explicit and temporally frequent monitoring of soil salinity [20]. Optical remote sensing, particularly using Landsat and Sentinel-2, has provided the foundation for numerous salinity monitoring studies by leveraging the reflectance characteristics of salt-affected soils in the visible, near-infrared (NIR), and short-wave infrared (SWIR) regions [21,22]. However, optical sensors are susceptible to atmospheric conditions and vegetation cover, which can obscure the soil’s spectral signature [23]. Synthetic Aperture Radar (SAR), such as Sentinel-1, offers a complementary solution, with its all-weather capability and sensitivity to soil moisture and surface structure, showing great potential for providing subsurface salinity information [24,25]. Furthermore, thermal infrared (TIR) remote sensing has recently gained attention due to its sensitivity to the distinct thermal emittance properties of salt-affected soils, providing an additional dimension for salinity discrimination [26]. The synergy of multi-source remote sensing data has been proven to enhance the accuracy of soil salinity prediction [25]. Several studies have successfully combined Sentinel-1 SAR and Sentinel-2 multispectral data, demonstrating superior performance over single-sensor approaches [27,28]. Moreover, the integration of ancillary environmental factors (e.g., topography, climate, soil properties) with spectral data can significantly improve model robustness by accounting for the complex drivers of salinization [29,30]. Ge et al. [30] reported a 5–8% accuracy increase by incorporating environmental variables alongside Sentinel-2 spectra. Concurrently, machine learning (ML) and deep learning (DL) approaches have been established as the prevailing methodology for characterizing the intricate non-linear dependencies of soil salinity on multi-spectral predictor variables. Among the prominent techniques, Random Forest (RF), XGBoost, and LightGBM have demonstrated particular efficacy [25,31,32]. Despite these advances, several challenges persist when applying these methodologies to specific, ecologically vulnerable regions.

Located in northwestern China, the Tarim River Basin represents a paradigmatic ecologically vulnerable zone, containing ~2.25 × 10^6^ hectares of saline–alkali land, accounting for 21.1% of the total saline–alkali land area in China [33]. These saline–alkali lands, which are fragmented and distributed in the desert–oasis transition zone of the Tarim River Basin, have enormous potential for economic utilization [2]. Serving as a major cotton cultivation base nationally, the Tarim Basin concurrently functions as China’s foremost premium-quality fiber production zone. As an important cotton-producing area in China, the Tarim River Basin is also the largest high-quality cotton-producing area [34]. Consequently, characterizing the spatial patterns and salinization dynamics of salt-affected soils within this basin constitutes a research priority. Wang et al. [35] adopted the traditional survey method of in-situ measurements of apparent electrical conductivity (ECa), involving electromagnetic induction (EMI), integrated environmental derivative variables, and an RF model, for generating spatially explicit representations of salt-affected soils across various vertical horizons at multiple depths. They verified the effectiveness of this method via a study of soil salinization in the Tarim River Basin. Liu et al. [36] mapped soil salinity at 10-m resolution in the Tarim River Basin, using soil samples and multi-source remote sensing, and they identified wind transport as the key driver of salt redistribution. Building on these efforts, the present study differs in three aspects: (i) ten machine learning algorithms are systematically compared under five multi-source feature scenarios rather than relying on a single model; (ii) Sentinel-1 polarimetric features are integrated with optical indices and environmental covariates; and (iii) SHAP-based interpretation is used to attribute model predictions to physically meaningful controls. These studies show that only by accurately monitoring dynamic soil salinization can we fully determine the status of saline–alkali land, assess its ecological impact, and formulate management strategies to enhance its agricultural productivity [9,35,37,38]. However, considering the basin’s vast expanse exceeding 1.02 × 10^6^ km^2^, it is difficult to investigate the regional saline–alkali land using traditional sampling methods. Thus, in this study we combined remote sensing and an actual field investigation to establish a robust model for inverting the distribution and salinization characteristics of saline–alkali land in the Tarim River Basin.

Spatially distributed sampling was conducted across the Tarim River Basin’s saline–alkali landscapes, generating a dataset of 147 soil samples subsequently analyzed for salt content via conductivity assays. By incorporating machine learning algorithms, we formulated a reliable soil salinity retrieval framework for the mainstream of the Tarim River by integrating multi-source remote sensing data (Sentinel-1, Sentinel-2) and environmental covariates. Our overall aims were to systematically evaluate and compare the performance of multi-sensor data combinations and multiple machine learning models for soil salinity retrieval in arid and hyper-arid regions. Specifically, we: (1) Identified and extracted remote sensing–sensitive indicators (e.g., salinity, vegetation, SAR indices) along with key environmental covariates applicable to the Tarim River Basin. (2) Utilized the Extreme Gradient Boosting (XGBoost) algorithm coupled with SHAP (SHapley Additive exPlanations) values to screen key features across different scenarios and subsequently constructed and validated a precise soil salinity retrieval model by comparing various multi-sensor data combinations and machine learning algorithms. (3) Analyzed the sensitivity of multi-source remote sensing data for salinity prediction and mapped the spatial distribution of soil salinity at high resolution. Our findings furnish empirical evidence for precision land management and ecological restoration in arid zones.

## 2. Research Area and Sampling

### 2.1. Study Area

**Figure 1:**
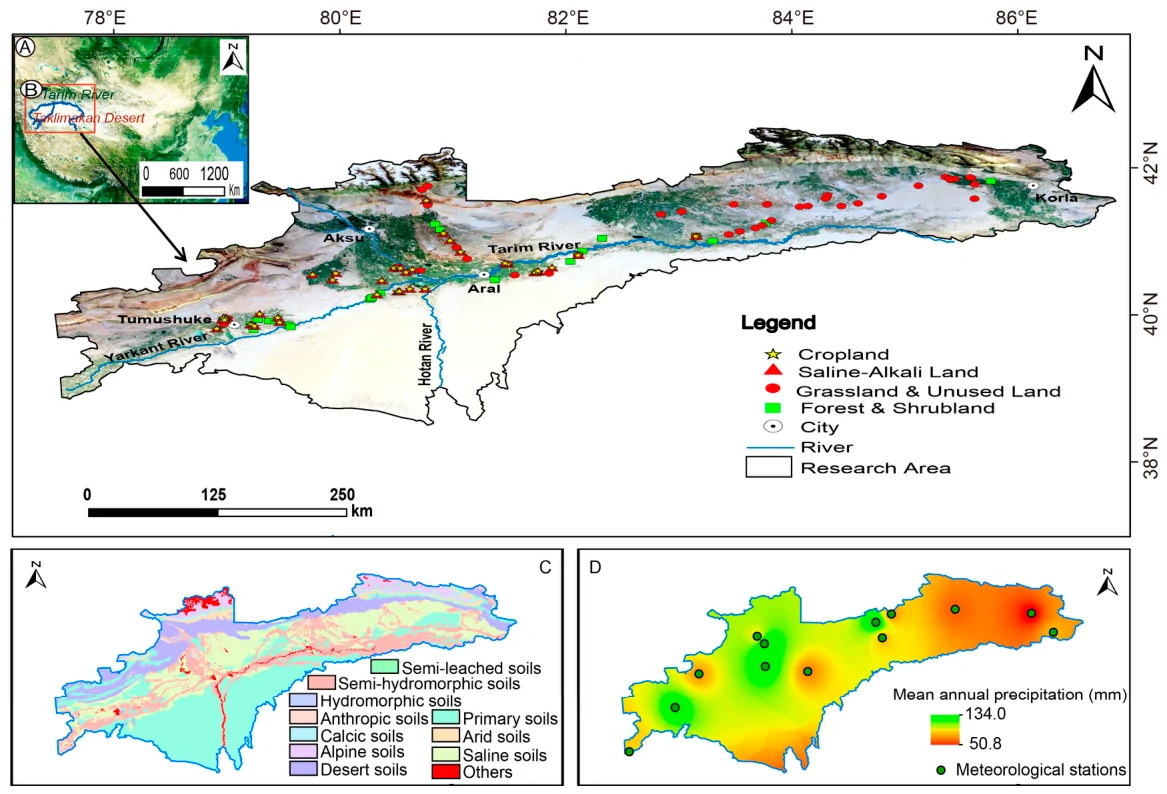
Overview of the study area: (**A**) location of the Tarim River Basin in northwestern China; (**B**) Distribution of the 147 sampling sites across the middle and upper courses of the Tarim River Basin, coloured by land-use type, together with the river network, major cities, and the basin boundary; the inset; (**C**) Soil-type distribution according to the Chinese soil genetic classification (anthropogenic, alpine, semi-leached, primary, calcic, semi-hydromorphic, arid, hydromorphic, desert, and saline soils, and others); (**D**) Mean annual precipitation (1963–2023) interpolated from China Meteorological Administration stations (dots) by inverse distance weighting.

Situated in the northern sector of the Tarim Basin within northwestern China (39°30′–42°00′ N, 77°00′–88°00′ E), the study area encompasses the middle and upper courses of the Tarim River (Figure 1A). With salt-affected soils occupying over 21.1% of the national total, this basin serves as an exemplar site for investigating saline–alkali dynamics [2,33]. The research area includes the alluvial plain to the south of the Tianshan Mountains and the oasis–desert area in the northern Taklimakan Desert, with an elevation range of 800–1500 m (Figure 1B). The climate is temperate and extremely arid, with a mean annual temperature of 7.8 °C, annual rainfall ranging from 50 to 135 mm (Figure 1D), and potential evapotranspiration between 2000 and 3000 mm [39]. Water resources are primarily recharged by snow and glacier melt from the Tianshan Mountains, Pamir Plateau, and Kunlun Mountains [40,41] (Figure 1B). Major soil types are dominated by desert soils, arid soils, and saline soils, with anthropogenic soils concentrated in the cultivated oases (Figure 1C); the surface soil (0–20 cm) has a salt content exceeding 87 g kg^−1^ in some areas [42]. The natural vegetation coverage is <30% in this area, dominated by xerophytic and halophytic shrub communities [43,44].

Since the 1960s, agricultural management in the basin has been dominated by cotton (*Gossypium hirsutum* L.) and winter wheat (*Triticum aestivum* L.) cultivation under flood irrigation practices, which has intensified groundwater extraction and secondary soil salinization; this agricultural abstraction has markedly diminished river discharge along the main stem and lowered groundwater tables in intensively irrigated areas [45,46]. Irrigation in the basin is sourced primarily from the Tarim River and its tributaries; the mineralization of irrigation water in the region commonly reaches 2–6 g L^−1^ [47], and conventional winter and spring leaching irrigation alone accounts for 55–65% of total water use (450–840 mm) [48]. Because flood irrigation is not matched by adequate drainage infrastructure, groundwater evaporation has been further intensified, aggravating secondary salinization. In contrast to the depleted water tables of the intensively pumped irrigated areas, shallow groundwater (<2 m depth) remains prevalent in the middle–lower reaches, where river and canal infiltration contributes more than 70% of total groundwater recharge according to local hydrogeological surveys [49,50]. Capillary rise from this shallow groundwater transports dissolved salts to the surface, where evaporative concentration leads to salt crust formation, particularly in areas with inadequate drainage [51,52]. The accumulated salts are dominated by chloride–sodium and sulfate–chloride types characteristic of the southern Xinjiang oases [47]. This hydrogeological setting creates a direct linkage between river proximity, groundwater depth, and surface soil salinity.

### 2.2. Field Sampling

**Figure 2:**
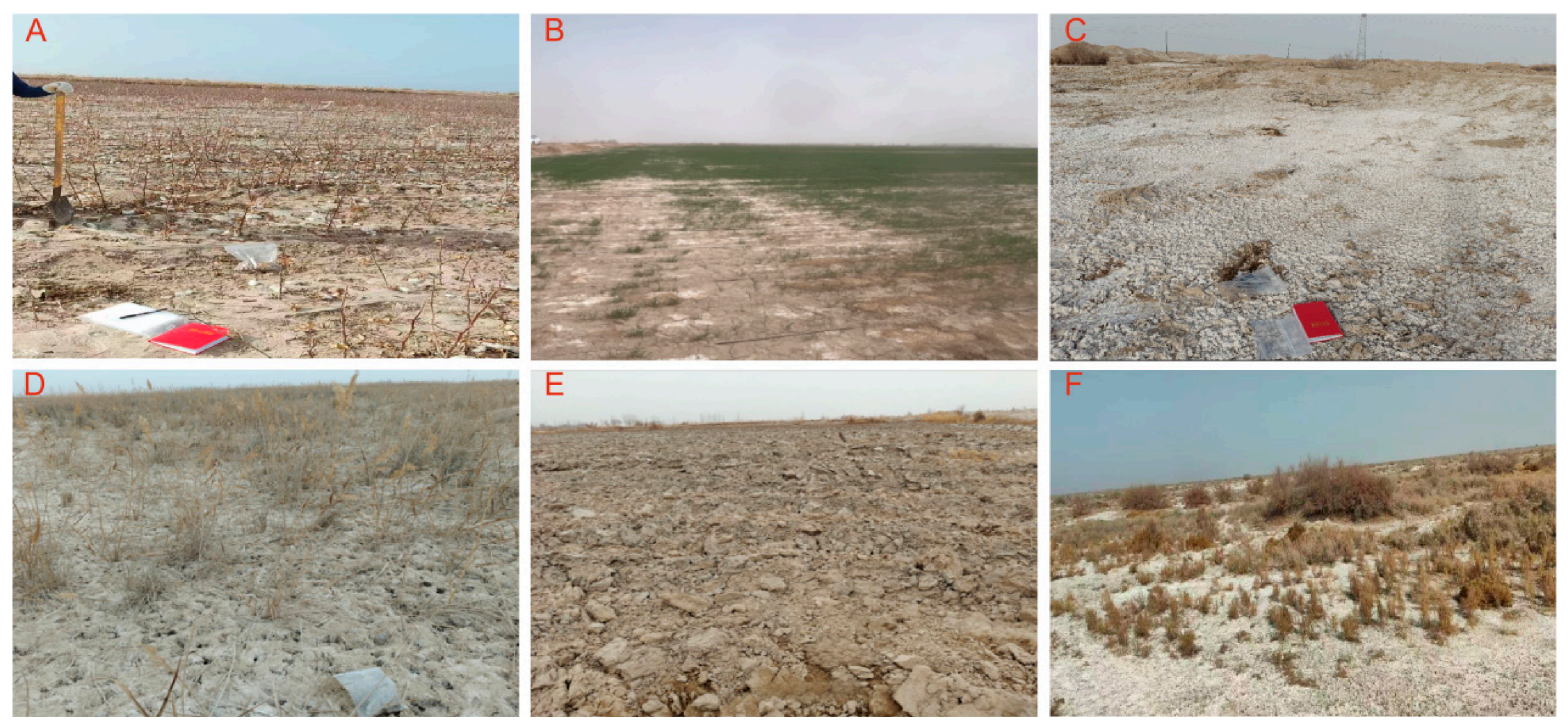
Examples of different landscape and saline–alkali land types in the Tarim River Basin. (**A**) Cropland (cotton), (**B**) cropland (wheat); (**C**) saline–alkali land, (**D**) grassland & unused land (natural grassland); (**E**) grassland & unused land (unused land); (**F**) forest and shrubland. These scenes illustrate the six dominant land-cover categories stratified during sampling.

Between September–November 2024 and April–May 2025, we performed field campaigns during bare-soil conditions, gathering 147 surface soil (0–5 cm) samples across the Tarim River Basin (Figure 1B) across various land-use categories (Figure 2). The 0–5 cm topsoil layer was targeted because it is most directly affected by evaporative salt accumulation and is the layer most directly sensed by optical and SAR remote sensing. These samples comprise cropland (42 samples, including cotton, wheat, maize fields, and orchards); saline–alkali land (42 samples, encompassing all lands significantly affected by salinity/alkalinity); grassland and unused land (42 samples, comprising natural grassland, wasteland, and unused land); forest and shrubland (21 samples, dominated by *Populus euphratica*, *Haloxylon ammodendron*, or shrubs). Approximately 500 g of topsoil was extracted per location with a hand shovel and immediately placed in pre-marked sampling bags. Positional data for all sampling locations were captured using a handheld GNSS unit (LT60H model). Sampling sites were stratified by land-use type and distributed along the river mainstream to capture the salinity gradient of the basin.

## 3. Materials and Methods

### 3.1. Workflow Overview

**Figure 3:**
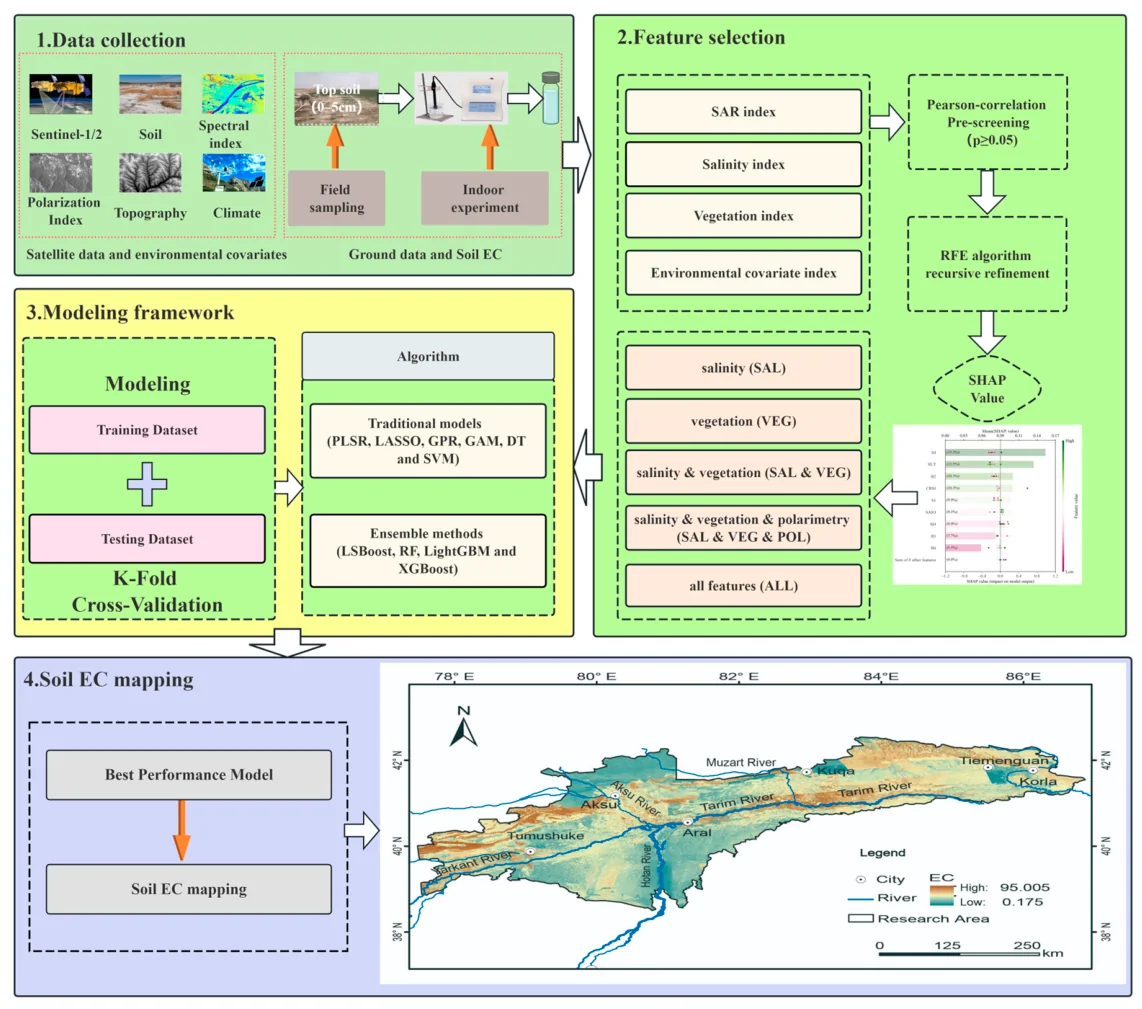
The workflow of this study, comprising four consecutive phases: (**1**) multi-source data collection; (**2**) three-stage feature selection (Pearson correlation pre-screening, recursive feature elimination, and SHAP interpretability ranking); (**3**) modelling under five feature-combination scenarios with ten machine learning algorithms; and (**4**) spatial mapping of soil electrical conductivity (EC).

This investigation was structured into four consecutive phases: (1) data collection, (2) feature selection, (3) modeling framework, and (4) soil electrical conductivity (EC) mapping (Figure 3). Detailed procedures are described below.

### 3.2. Data Collection

Multi-source datasets were integrated, including Sentinel-1/2 satellite imagery [53,54], topographic data, and climate variables. Field sampling was conducted to collect topsoil samples (0–5 cm), followed by laboratory experiments. Soil electrical conductivity (EC_1:5_) was employed as the salinity index, following USDA guidelines [55,56], and measured using a calibrated conductivity meter at 25 °C on a 1:5 soil–water extract to obtain ground reference data. Sentinel-2 imagery was atmospherically corrected using the Sen2Cor_v2.10 processor; all spectral and polarimetric features were resampled to a 10 m resolution.

Sentinel-1 Level-1 Ground Range Detected (GRD) images were acquired in Interferometric Wide (IW) swath mode with dual polarization (VV/VH) on an ascending orbit at incidence angles of approximately 33–43°, during the bare-soil periods of September–November 2024 and April–May 2025. In SNAP 8.0 (European Space Agency, Paris, France), the scenes were radiometrically calibrated, speckle-filtered using a Refined Lee filter (7 × 7 window), and terrain-corrected by Range-Doppler RTC with the SRTM 30-m DEM, then converted to backscatter coefficients (σ^0^, dB), resampled to 10-m resolution, and co-registered with Sentinel-2 imagery; the full processing chain is documented in Supplementary Information S1.2.

Mean annual temperature and precipitation data were obtained from 13 meteorological stations of the China Meteorological Administration (CMA) within the study area. Data covered the period 1963–2023 and were interpolated to 30-m rasters using inverse distance weighting (IDW) in ArcGIS 10.8 (Esri, Redlands, CA, USA). Potential evapotranspiration (PET, 2000–3000 mm yr^−1^) was estimated using the FAO-56 Penman–Monteith method based on the same station data. The source, station count, and period of record for all climatic variables are documented in Supplementary Information S1.3.

### 3.3. Feature Selection

This was based on four categories of indices: SAR indices, salinity indices, vegetation indices, and environmental covariate indices. Feature selection was performed via a three-step procedure: First, Pearson correlation pre-screening was applied to remove features with *p* ≥ 0.05 (correlation with EC), retaining 49 variables. Second, we implemented recursive feature elimination (RFE) with Random Forest as the base learner, iteratively discarding the lowest-importance predictors while tuning through 10-fold cross-validation, ultimately retaining 42 key variables. Third, SHAP (SHapley Additive Explanations) values were calculated to quantify feature contributions and identify the most relevant variables. Consequently, five distinct feature scenarios were constructed for subsequent modeling: salinity (SAL), vegetation (VEG), salinity & vegetation (SAL & VEG), salinity & vegetation & polarimetry (SAL & VEG & POL), and all features (ALL). All indices and covariates used in this study are defined in the Supplementary Information: the 13 SAR indices and polarimetric transformations (VV, VH, and 11 derived combinations) are listed in Table S1 and defined explicitly, in the order of Table S1, in the accompanying note; the 26 salinity indices and 28 vegetation indices are given with formulas and references in Table S2; and the 16 environmental covariates (topographic indices plus MAT and MAP) are listed in Table S3 and described in Section S1.3.

### 3.4. Modeling Framework

We randomly divided the data into training (80%, 118 samples) and validation (20%, 29 samples) partitions. Within the training set, 10-fold cross-validation was employed for model selection and hyperparameter optimization. The optimal model was subsequently evaluated on the held-out testing set. The performance of two model categories was compared: (i) Traditional models including partial least squares regression (PLSR), least absolute shrinkage and selection operator (LASSO), Gaussian process regression (GPR), generalized additive model (GAM), decision tree (DT), and support vector machine (SVM); and (ii) ensemble methods including Least Squares Boosting (LSBoost), RF, LightGBM, and Extreme Gradient Boosting (XGBoost). Model performance was evaluated using the coefficient of determination (R^2^), root mean square error (RMSE), and the mean absolute error (MAE). The algorithm exhibiting the best predictive performance was selected as the optimal model. All data processing and modeling were conducted in MATLAB R2025a (The MathWorks, Natick, MA, USA); topographic indices were derived in SAGA GIS 7.4.0 (University of Hamburg, Hamburg, Germany), and mapping was performed in ArcGIS 10.8 (Esri, Redlands, CA, USA). To assess the reproducibility of the reported accuracies, the random 80%/20% train–test split was repeated 100 times with different random seeds, and the best-performing models were retrained under the ALL scenario for each split; in addition, the uncertainty of R^2^, RMSE, and MAE was quantified using 95% confidence intervals (CIs) derived from 1000 bootstrap resamples of the test-set predictions in each split (Section 4.3).

### 3.5. Soil EC Mapping

The best-performing model was applied to the entire study area to generate a map of the spatial distribution of soil salinity, visually illustrating the spatial variability and gradient patterns of EC levels.

## 4. Results

### 4.1. Descriptive Statistics and Spatial Heterogeneity of Soil Salinity

**Figure 4:**
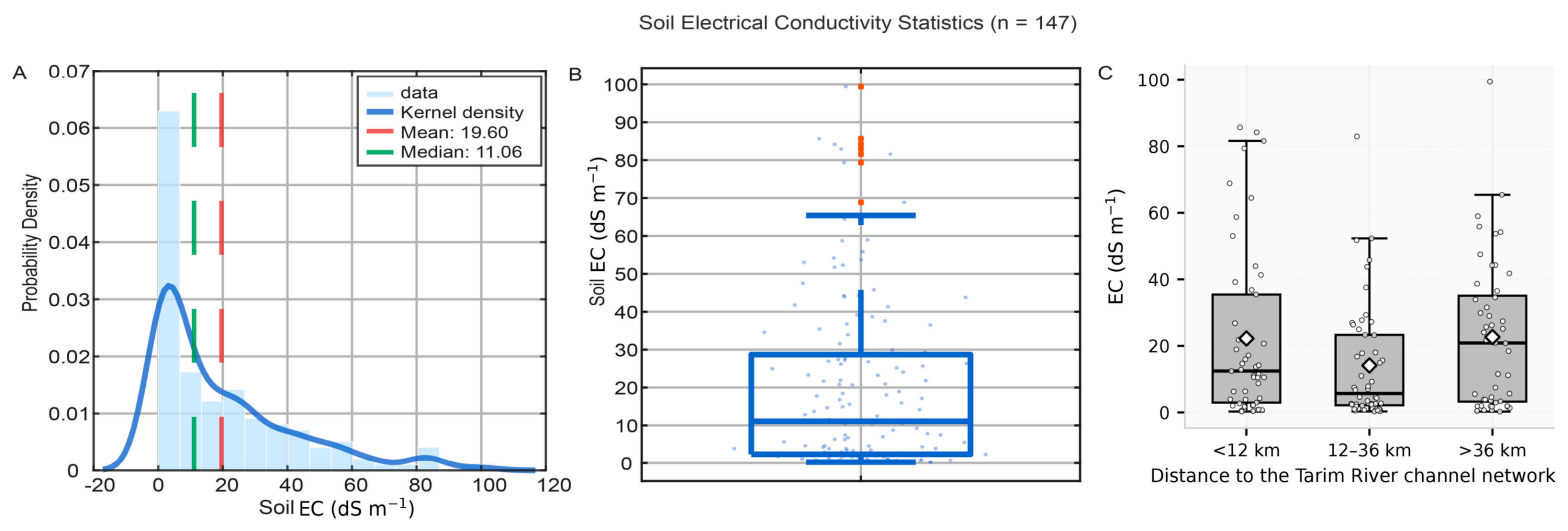
Probability density distribution of soil electrical conductivity (EC) values (**A**), box plot of the EC values (**B**), and EC grouped by distance to the nearest channel of the Tarim River network (<12 km, 12–36 km, >36 km; *n* = 49, 50, and 48) (**C**). The strongly right-skewed distribution (**A**) and the comparable salinity levels of the near-channel and distal groups (**C**) jointly reflect the combined association of soil salinity with shallow groundwater, irrigation–drainage practices, and distance to the river channel.

Soil electrical conductivity (EC), a key indicator of soil salinity, exhibited substantial spatial variability across the study area in the hyper-arid Tarim River Basin (Table 1 and Supplementary Table S4). Measured EC values ranged from 0.24 to 99.43 dS m^−1^. The considerable disparity between mean (19.60 dS m^−1^) and median (11.06 dS m^−1^) EC values, coupled with the high coefficient of variation (113.04%), reflects a strongly right-skewed distribution (Figure 4A). The presence of outliers (4.8% of samples) in a box plot (Figure 4B) visually confirmed the occurrence of localized high-salinity hotspots within a generally heterogeneous salinity landscape.

To examine the spatial association between river proximity and soil salinity, we calculated the Euclidean distance from each sampling point to the nearest channel of the Tarim River network (the main stem together with the Aksu, Yarkand, and Hotan source rivers) using ArcGIS 10.8. Sampling points were divided into three groups of approximately equal sample size by distance (<12 km, 12–36 km, and >36 km). Box-plot comparison (Figure 4C) shows that the lowest EC values occur at intermediate distances from the channel network, whereas high salinity occurs both in the near-channel corridor and in the distal downstream plain. This non-monotonic pattern suggests that basin-scale soil salinity is jointly associated with channel proximity, irrigation–drainage management, and land-use type, rather than with river distance alone.

### 4.2. Feature Selection for Predictive Modeling

#### 4.2.1. Correlating Surface Attributes with Measured EC

**Figure 5:**
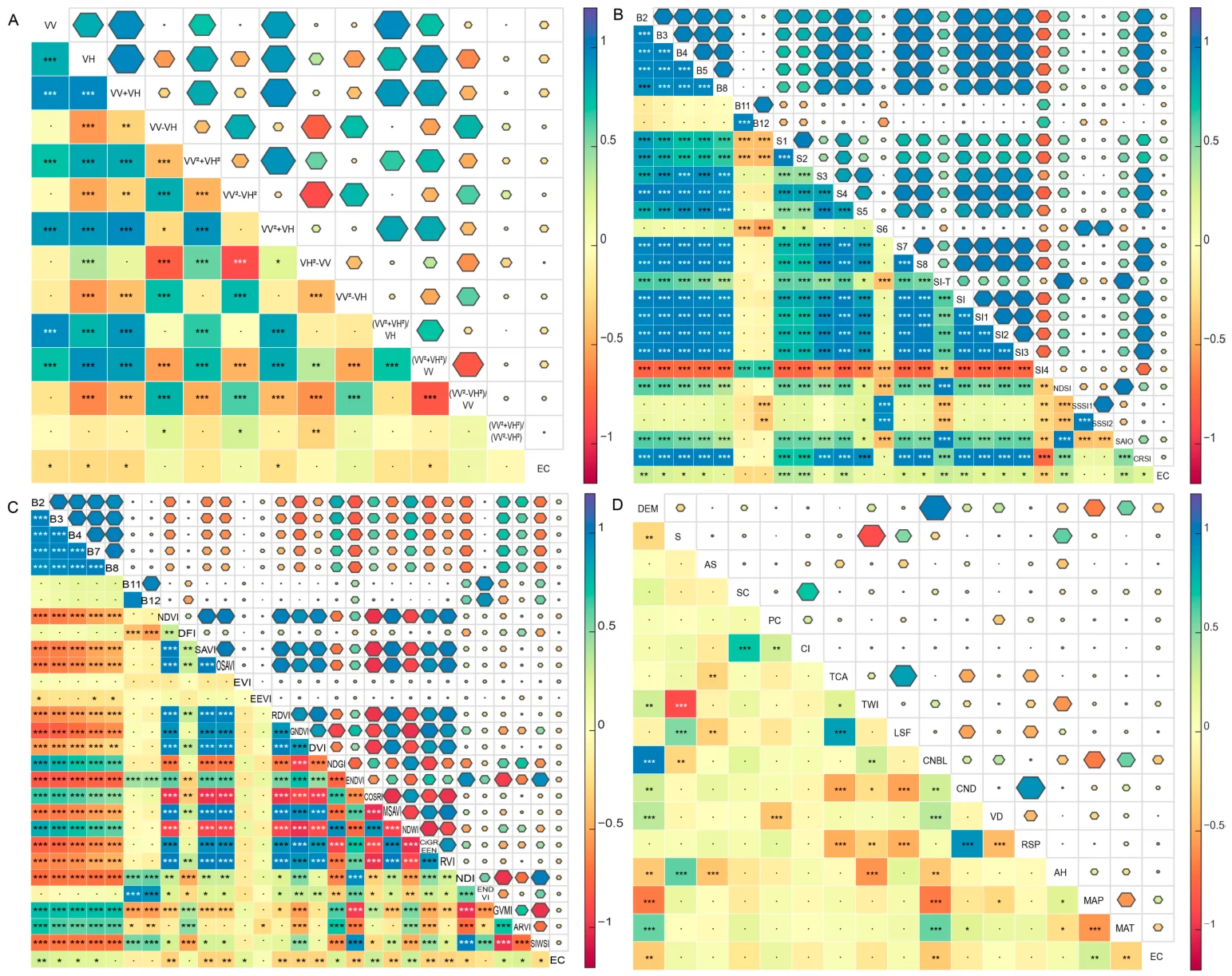
Pearson correlation coefficients between measured electrical conductivity (EC) values and the SAR index (**A**), salinity index (**B**), vegetation index (**C**), and environmental covariate index (**D**). * Significant correlation at *p* < 0.05; ** highly significant correlation at *p* < 0.01; *** extremely significant correlation at *p* < 0.001;· not significant (*p* ≥ 0.05). Salinity indices were predominantly positively correlated with EC, vegetation indices were negatively correlated, and only topographic and climatic covariates among the environmental variables exhibited significant correlations, indicating the combined spectral–climatic controls on soil salinity.

Pearson correlation analysis was performed on 83 candidate parameters (listed in Supplementary Table S1–S3). Results showed that 24 parameters were highly correlated with EC (*p* < 0.01), and 23 were significantly correlated (*p* < 0.05) (Figure 5). Among 13 SAR-derived metrics, 5 demonstrated statistically significant EC linkages (*p* < 0.05) (Figure 5A). Polarimetric transformations effectively reduced noise-related artifacts in surface scattering responses, thereby enhancing the associations between SAR indices and EC. Among the salinity indices, seven showed highly significant EC correlations (*p* < 0.01) (Figure 5B). Specifically, Salinity Index 1 (S1) and Salinity Index 2 (S2) exhibited the highest degree of positive correlations, with corresponding Pearson coefficients of 0.358 (S1) and 0.352 (S2). In contrast, vegetation indices were negatively correlated with EC; of those analyzed, 13 were highly significantly correlated (*p* < 0.01) (Figure 5C). For environmental covariates, only the Digital Elevation Model (DEM), Channel Network Base Level (CNBL), mean annual temperature (MAT), and mean annual precipitation (MAP) showed highly significant correlations (*p* < 0.01) with EC (Figure 5D).

#### 4.2.2. Recursive Feature Elimination (RFE) and Predictor Importance

**Figure 6:**
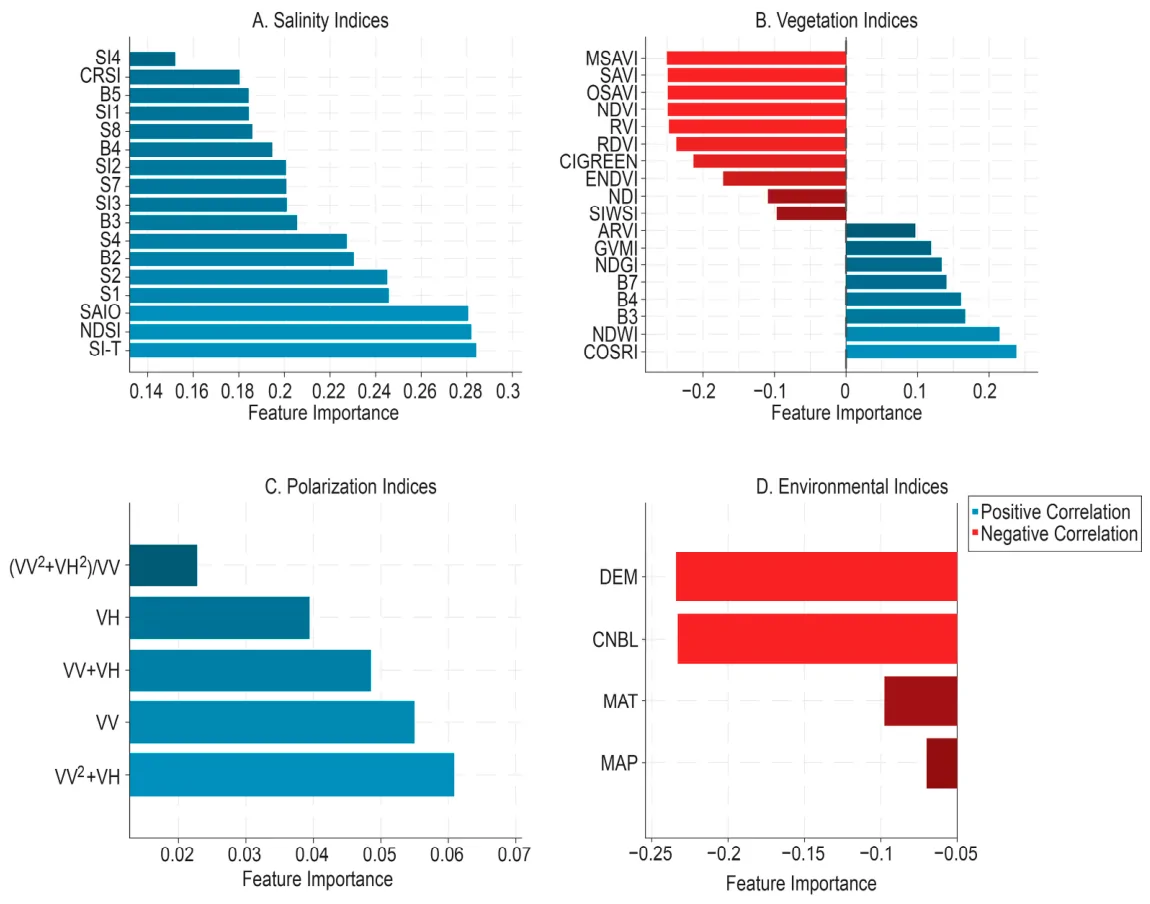
Feature-importance ranking for saline–alkali land retrieval using recursive feature elimination (RFE): (**A**) salinity index feature importance; (**B**) vegetation index feature importance; (**C**) polarimetric index feature importance; (**D**) environmental index feature importance. Within each category, the highest-importance indices (SI-T, NDSI, and SAIO for salinity; MSAVI, SAVI, OSAVI, and NDVI for vegetation; DEM and CNBL for environmental covariates) were retained, whereas the limited importance of the polarimetric indices indicates that SAR information alone is insufficient for independent salinity retrieval under the complex surface conditions of the study area.

Following recursive feature elimination, the retained predictors across the four categories exhibited distinct importance patterns (Figure 6). Salinity indices demonstrated positive importance scores, underscoring their direct mechanistic linkage to soil salt content (Figure 6A). Notably, SI-T, NDSI, and SAIO emerged as the three most influential indicators, consistent with their theoretical ability to capture salt-induced spectral alterations [57]. In contrast, vegetation indices showed distinctly negative importance, clearly reflecting the inhibitory effect of salt stress on plant growth [58]. MSAVI, SAVI, OSAVI, and NDVI were identified as the most important within this category (Figure 6B), confirming their effectiveness as indirect proxies for salinization. Polarimetric indices derived from Sentinel-1 SAR data made only a limited contribution (Figure 6C), suggesting that polarimetric information alone is insufficient for reliable and independent salinity retrieval in the complex surface conditions of the study area. The integration of environmental factors demonstrated landscape-scale controls, where DEM and CNBL emerged as the most important predictors, suggesting topographic and hydrogeological dominance in soil salinity distribution [59] (Figure 6D). The RFE analysis successfully achieved intra-category feature prescreening, providing a refined feature set composed of the most representative predictors from each category for model construction.

#### 4.2.3. Global Feature Importance Analysis Based on SHAP

SHAP (SHapley Additive exPlanations) analysis characterized how feature contributions are organized in the spatial retrieval of soil salinity along the Tarim River mainstream. Distinct from the intra-category feature rankings identified by RFE, the model-wide feature contributions exhibited a clear progression from micro-scale spectral characteristics to macro-scale environmental drivers, reflecting statistical associations rather than a mechanistic decision process.

**Figure 7:**
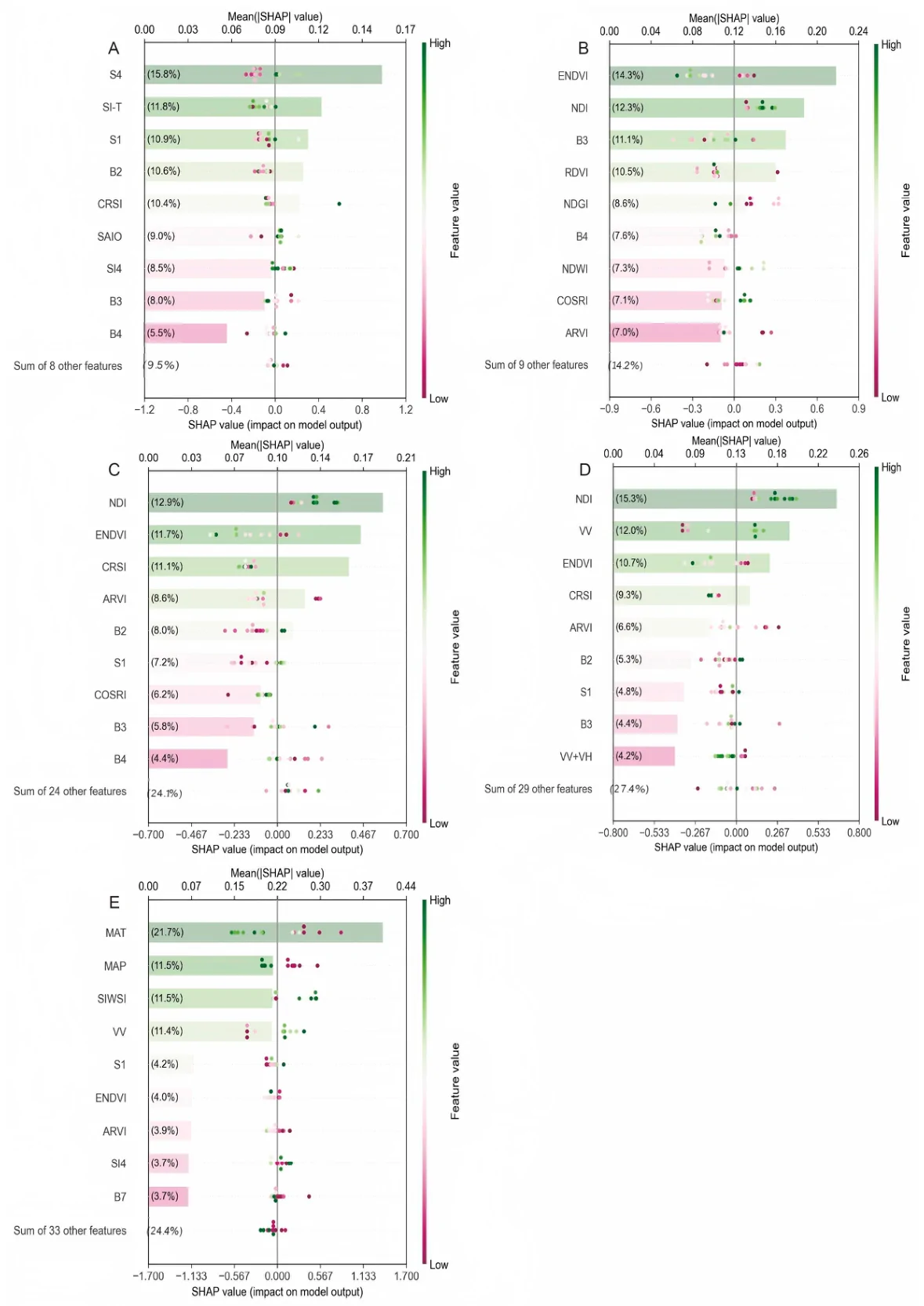
SHAP summary plots showing feature importance across five feature combination scenarios: (**A**) salinity indices, (**B**) vegetation indices, (**C**) salinity + vegetation indices, (**D**) salinity + vegetation + SAR polarization features, and (**E**) all features including environmental variables. Features are ranked by mean absolute SHAP value (upper x-axis). Each point represents an individual sample (*n* = 147), with color indicating feature value (green: high; purple: low). Percentages denote relative contribution to total feature importance.

At the salinity-index-dominated stage (Figure 7A), predictors within this category—including both spectral indices and their constituent visible bands—drove the decision process. S4 (|SHAP| = 15.8%), SI-T (11.8%), and S1 (10.9%) ranked as the top three contributors, underscoring their direct mechanistic linkage to soil salt content. The original spectral bands showed moderate positive contributions, with B2 (10.6%), B3 (8.0%), and B4 (5.5%) retaining predictive power despite the dominance of derived indices. Secondary salinity proxies including CRSI (10.4%), SAIO (9.0%), and SI4 (8.5%) collectively accounted for 27.9% of model variance, while the remaining eight features contributed 9.5%.

Within the vegetation-index category (Figure 7B), which encompassed both vegetation-specific metrics and their spectrally sensitive bands, several features demonstrated inverse relationships with salinity predictions. ENDVI (14.3%) and NDI (12.3%) ranked as the top contributors with the largest magnitude of SHAP values, their negative components reflecting the inhibitory effect of salt stress on plant growth [52]. Notably, bands B3 (11.1%) and B4 (7.6%) ranked among the top contributors within this category, suggesting that raw spectral information in the green and red regions complements vegetation metrics in capturing salt-stress signals. Additional indices including the water-related NDWI (7.3%), the salinity-vegetation response index COSRI (7.1%), and the vegetation index ARVI (7.0%) made consistent negative contributions. The nine remaining features collectively contributed 14.2%.

In the integrated optical stage (Figure 7C), which combined salinity and vegetation indices, the vegetation stress indicators were the most important. NDI (12.9%) and ENDVI (11.7%) surpassed direct salinity indices, while CRSI (11.1%) and ARVI (8.6%) made substantial contributions. The blue band B2 (8.0%) remained the dominant spectral band, whereas S1 (7.2%) decreased in relative importance compared to the salinity-only stage, indicating partial redundancy when vegetation stress signals were incorporated. COSRI (6.2%), B3 (5.8%), and B4 (4.4%) made moderate contributions, and the 24 remaining features contributed 24.3%.

With the incorporation of Sentinel-1 SAR polarization features (Figure 7D), VV polarization emerged as a significant predictor (12.0%), with mean |SHAP| values indicating positive contributions across samples. NDI (15.3%) reached its peak importance at this stage, followed by ENDVI (10.7%) and CRSI (9.3%). The cross-polarization combination VV + VH contributed 4.2%. Despite SAR integration, vegetation indices maintained collective dominance (NDI + ENDVI = 26.0%), while the 29 remaining features contributed 27.4%—notably including B4 which decreased in relative importance compared to the pure spectral stage (Figure 7B).

Finally, upon inclusion of environmental covariates (Figure 7E), the full model exhibited a marked reorganization of feature importance. MAT (21.7%) exhibited the highest mean |SHAP| value among all features across all stages. Water availability emerged as a critical theme, with MAP (11.5%) and the water stress index SIWSI (11.5%) showing comparable contributions to model predictions. VV polarization maintained moderate importance (11.4%), capturing surface roughness associated with salt crusts. Notably, the importance of optical indices decreased in this final stage (S1: 4.2%; ENDVI: 4.0%; ARVI: 3.9%), while the short-wave infrared band B7 (3.7%) and SI4 (3.7%) contributed only marginally, likely reflecting moisture-related spectral signals. The 33 remaining features contributed 24.4%. This shift from spectral to climatic dominance suggests that while optical-SAR features capture local-scale spectral variations, environmental variables govern the broad-scale salinity distribution patterns along the Tarim River.

### 4.3. Model Performance Comparison

Systematic evaluation of ten machine learning models across five feature combination scenarios identified the optimal strategy for remote sensing retrieval of saline–alkali land (Table 2). RF achieved optimal performance in the ALL scenario (full feature set including environmental variables) (R^2^ = 0.73, RMSE = 3.58 dS m^−1^, MAE = 1.89 dS m^−1^), consistent with established findings regarding RF’s robustness in handling complex, high-dimensional environmental datasets [60]. The superior performance of ensemble methods was evident across all scenarios, with XGBoost and LSBoost also demonstrating strong predictive capabilities (ALL scenario: XGBoost R^2^ = 0.66, RMSE = 4.07 dS m^−1^, MAE = 1.82 dS m^−1^; LSBoost R^2^ = 0.68, RMSE = 3.89 dS m^−1^, MAE = 2.10 dS m^−1^), whereas traditional algorithms including PLSR, Lasso, DT, and SVM exhibited consistently poor generalization, with R^2^ values below 0.40 in most scenarios, occasionally approaching zero, indicating inadequate capacity for capturing non-linear relationships in heterogeneous saline–alkali environments [61].

Progressive feature integration revealed scenario-specific algorithmic optima (Table 2). In the SAL scenario utilizing salinity indices exclusively, Gaussian Process Regression (GPR) demonstrated superior accuracy (R^2^ = 0.62, RMSE = 3.99 dS m^−1^, MAE = 2.05 dS m^−1^), highlighting its effectiveness in managing limited feature dimensions with Gaussian-distributed uncertainties [62]. Conversely, RF excelled in the VEG scenario (R^2^ = 0.62, RMSE = 3.98 dS m^−1^, MAE = 2.53 dS m^−1^), suggesting optimal processing of spectral vegetation indicators. The SAL & VEG scenario witnessed XGBoost achieving marginal superiority (R^2^ = 0.64, RMSE = 3.63 dS m^−1^, MAE = 2.03 dS m^−1^), consistent with previous comparative studies of ensemble methods [63]. The SAL & VEG & POL scenario saw RF and XGBoost attaining comparable optimal performance (R^2^ = 0.73 for both; RMSE = 3.48 vs. 3.52 dS m^−1^; MAE = 2.10 vs. 1.68 dS m^−1^ for XGBoost), with XGBoost showing superior mean error precision despite nominally equivalent R^2^. Notably, in the fully integrated ALL scenario, RF exclusively maintained this accuracy level (R^2^ = 0.73, RMSE = 3.58 dS m^−1^, MAE = 1.89 dS m^−1^), whereas XGBoost exhibited performance degradation (R^2^ = 0.66, RMSE = 4.07 dS m^−1^, MAE = 1.82 dS m^−1^), underscoring RF’s superior robustness to high-dimensional input and potential feature redundancy, despite XGBoost achieving marginally lower MAE in the full feature space (1.82 vs. 1.89 dS m^−1^).

These results reveal divergent responses to feature dimensionality among algorithms. RF maintained stable accuracy when inputs expanded from three to four domains (SAL & VEG & POL to ALL), while XGBoost and LSBoost showed reduced performance with the full feature set relative to the optical-SAR fusion scenario. GPR accuracy tended to decrease beyond single-domain inputs. This divergence suggests that optimal model selection requires matching algorithmic capacity to feature space dimensionality rather than relying solely on overall performance rankings.

To evaluate reproducibility, we repeated the train/test split 100 times using different random seeds (seeds 1–100, maintaining the 80%/20% ratio) and retrained the RF and XGBoost models under the ALL scenario in each split. Model performance remained stable across the 100 resplits (Table 3): RF achieved R^2^ = 0.70 ± 0.09, RMSE = 3.81 ± 0.15, and MAE = 1.62 ± 0.12, whereas XGBoost achieved R^2^ = 0.66 ± 0.10, RMSE = 3.89 ± 0.16, and MAE = 1.68 ± 0.13 (mean ± SD). The coefficient of variation of R^2^ was below 16% for both models (12.9% for RF and 15.2% for XGBoost), and the feature-importance rankings were highly consistent across splits (Kendall’s τ = 0.78 for RF and 0.72 for XGBoost, *p* < 0.001), confirming that the reported accuracy is reproducible and not an artifact of a particular random partition.

To quantify uncertainty, 95% confidence intervals (CIs) were computed for all key accuracy metrics from 1000 bootstrap resamples of the test-set predictions in each split; within every bootstrap iteration, the test-set predictions were resampled with replacement, R^2^, RMSE, and MAE were recalculated, and the 2.5th and 97.5th percentiles were taken (Table 3). The resulting intervals were narrow and symmetric around the point estimates for both models (Table 3); for example, RF (ALL) achieved R^2^ = 0.70 [95% CI: 0.614–0.782]. The bias-corrected and accelerated (BCa) intervals were nearly identical to the percentile intervals (Table 3), indicating that the bootstrap estimates are stable and reliable. Because these intervals reflect test-set sampling variability within individual splits, the significance of the between-model difference was additionally assessed from the 100 paired splits: RF exceeded XGBoost by 0.045 in R^2^ on average (95% CI: 0.038–0.052; paired *t*-test, *p* < 0.001), confirming that the performance differences discussed above are statistically meaningful.

**Figure 8:**
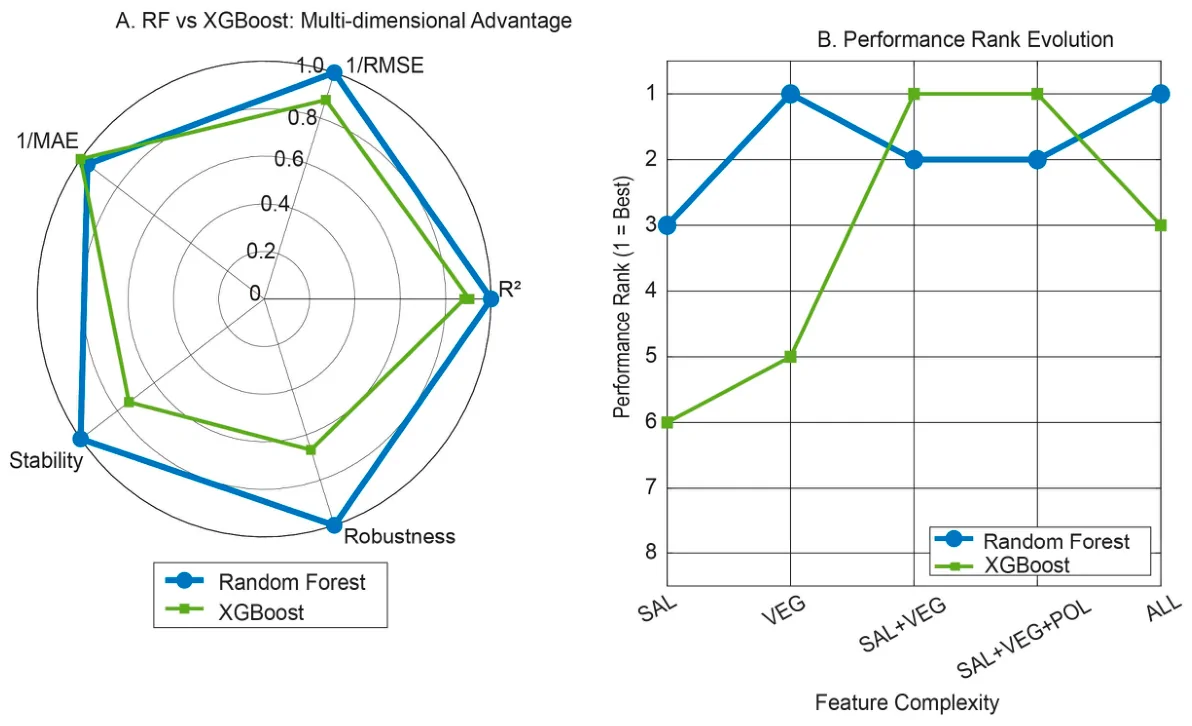
Comparative performance evaluation of Random Forest and XGBoost algorithms under varying feature combination scenarios: (**A**) multi-dimensional advantage analysis across feature combinations (comparison of inverse error metrics (1/RMSE, 1/MAE), stability, and robustness); (**B**) performance ranking of RF and XGBoost as feature combination complexity increases.

For the prominent ensemble models (RF and XGBoost), Figure 8 provides further insight into how the complexity of feature combinations correlates with model performance. Multi-dimensional evaluation indicated that while both models achieved comparable goodness-of-fit (R^2^) and mean absolute error (MAE), RF exhibited superior predictive precision as measured by RMSE. Most notably, RF outperformed XGBoost in stability and robustness indices (Figure 8A), suggesting stronger generalization capability under data perturbations across varying feature scenarios.

Performance ranking evolution across complexity gradients revealed divergent trajectories (Figure 8B). RF maintained consistently high rankings across all scenarios, with minimal volatility from low-dimensional to high-dimensional inputs. In contrast, XGBoost exhibited substantial fluctuations, performing poorly in the single-domain (SAL) setting but improving markedly with added vegetation and polarization features (SAL & VEG, SAL & VEG & POL), before decreasing again when environmental variables were introduced (ALL). These contrasting patterns indicate that RF maintains reliable performance regardless of input dimensionality, whereas XGBoost performance is more sensitive to specific feature combinations.

### 4.4. Soil EC Mapping and Its Spatial Pattern

**Figure 9:**
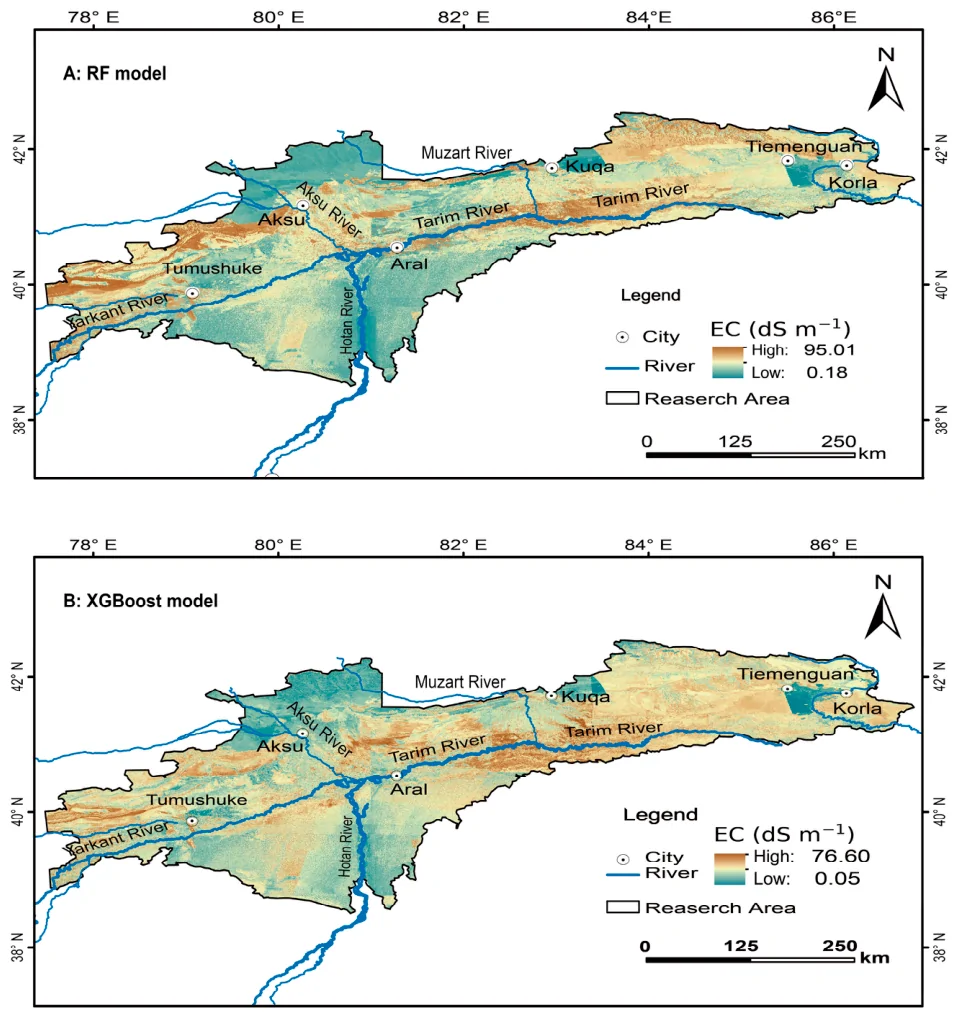
Maps of predicted soil salinity (EC, dS m^−1^) in the hyper-arid Tarim River Basin from the optimal model (Random Forest, RF) and the suboptimal model (Extreme Gradient Boosting, XGBoost).

Based on the optimal model rankings (Table 2), RF and XGBoost were selected to generate spatial distribution maps of soil EC for the Tarim River Basin (Figure 9). These maps were used to evaluate the spatial heterogeneity of salinization patterns and validate model performance against field measurements (Table 1).

Both models produced similar spatial distribution characteristics of soil EC (Figure 9). Salinity exhibited distinct longitudinal zonation, with higher values concentrated in the middle and lower basin reaches and lower values in the upper reaches. Topographically, elevated salinity characterized alluvial plains and river terraces, while depressed levels marked mountainous areas and desert–oasis transition zones. In the southern basin (~40° N), salinity displays a pronounced latitudinal zonation aligned with Tarim River tributary flow directions, whereas the northern mountain–plain transition zone exhibits scattered, patchy salinization patterns without clear directional organization.

## 5. Discussion

### 5.1. Methodological Reliability and Algorithmic Complementarity in Hyper-Arid Environments

Previous regional-scale soil salinity mapping in arid regions relied mainly on single-algorithm approaches, particularly RF, emphasizing its robustness with high-dimensional data [35,60]. While XGBoost has shown promise in structured datasets, systematic comparisons across varying feature dimensionality within hyper-arid landscapes remain scarce [61,63]. The potential for algorithmic complementarity, where different models excel in distinct tasks, has been overlooked, with few studies exploiting RF and XGBoost synergistically for regional-scale mapping.

**Figure 10:**
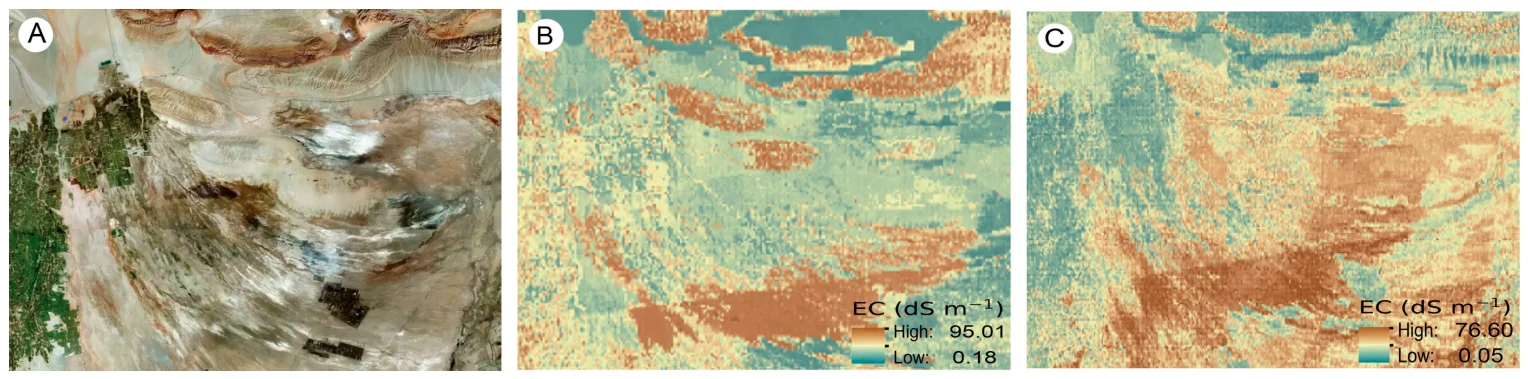
Detailed comparison of a typical sub-region. (**A**) Satellite image (Sentinel-2A, May 2025); (**B**) EC retrieval results using the RF model; (**C**) EC retrieval results using the XGBoost model.

Our comparative analysis of 147 field samples (CV = 113.04%) reveals distinct algorithmic niches. RF maintained stable performance across all feature scenarios (R^2^ = 0.60–0.73), achieving optimal accuracy with the full multi-source integration (R^2^ = 0.73, RMSE = 3.58 dS m^−1^, MAE = 1.89 dS m^−1^). Crucially, RF preserved the full dynamic range of soil salinity (measured: 0.24–99.43 dS m^−1^; predicted: 0.18–95.01 dS m^−1^), accurately capturing extreme hotspots. In contrast, XGBoost was sensitive to feature complexity: performance peaked with three data sources (SAL & VEG & POL: R^2^ = 0.73) but degraded with full environmental integration (ALL: R^2^ = 0.66), reducing the predicted maximum to 76.60 dS m^−1^. Sub-region analysis (Figure 10) further illuminates these divergent behaviors. In the piedmont alluvial plain–oasis ecotone, RF produced continuous EC gradients with smooth transitional zones between bare soil and vegetation (Figure 10B), whereas XGBoost generated sharper classification boundaries with higher spatial fragmentation (Figure 10C). This suggests that RF excels in continuous gradient mapping and extreme value identification, while XGBoost provides superior performance for binary saline vs. non-saline delineation at ecotone boundaries. The combined 33.2% contribution of MAT and MAP reported above represents a statistical association derived from the model’s feature-importance analysis rather than a causal attribution; climatic, spectral, and SAR variables jointly constitute the statistically significant controls identified by the SHAP analysis (Figure 7E).

These performance differences stem from fundamental algorithmic mechanisms interacting with the high-heterogeneity environment (CV = 113.04%). RF’s bootstrap aggregation strategy reduces variance without biasing predictions toward the mean, preserving the full dynamic range essential for capturing extreme salinity values (outliers comprising 4.8% of samples). Conversely, XGBoost’s gradient boosting mechanism optimizes a regularized objective function that inherently penalizes outliers, resulting in compressed prediction ranges but enhanced boundary detection. SHAP analysis indicated that both models produce physically consistent feature contributions: the decision hierarchy prioritizes climatic drivers (MAT + MAP, 33.2%), refines predictions via SAR properties (VV), and incorporates vegetation stress indicators (ENDVI/NDI) [64,65]. This alignment between statistical importance and actual biophysical processes—climate governing the water balance, SAR detecting surface roughness, vegetation reflecting osmotic stress—suggests that the models capture hydrologically meaningful constraints rather than purely spurious correlations.

### 5.2. Physical Mechanisms Governing C-Band Backscatter over Saline Soils

The backscatter coefficient (σ^0^) of saline soils is governed jointly by surface roughness and complex dielectric permittivity. Salt accumulation increases both the real (ε′) and imaginary (ε″) components of the soil dielectric constant—the latter through ionic conduction—and at C-band frequencies (4–8 GHz) the real part of the dielectric constant of saline soil can reach three to five times that of dry soil, enhancing σ^0^ by 2–8 dB [66,67,68].

Surface roughness constitutes the second control. Salt crusts and efflorescences alter the surface roughness spectrum, with typical RMS heights of 0.5–2.0 cm and correlation lengths of 5–15 cm; relative to the C-band wavelength (~5.6 cm), these wavelength-scale irregularities enhance diffuse scattering at the moderate incidence angles of this study (33–43°), thereby raising VV backscatter [66,69].

A weak volume-scattering contribution may additionally arise where salt accumulates within the 0–5 cm surface layer in high-salinity (>20 dS m^−1^) areas. Given the low soil moisture content (<10%) of the basin, dielectric variations are driven primarily by salinity rather than moisture. These mechanisms explain why the VV polarization contributed 12.0% to the full model and why the SAR signal is physically consistent with the optical and climatic controls described above: the two sensors capture complementary properties of the salt crust—Sentinel-2 its mineral composition and colour (particularly in the SWIR), Sentinel-1 its dielectric and geometric changes.

Three aspects of the experimental design constrain the moisture confounding of this dielectric mechanism. First, all Sentinel-1 scenes were acquired during bare-soil periods (September–November 2024 and April–May 2025), when soil moisture is low and spatially stable (precipitation 50–135 mm yr^−1^; potential evapotranspiration 2000–3000 mm yr^−1^), minimizing moisture-induced variability of the dielectric permittivity. Second, moisture-sensitive optical indices (SIWSI and NDWI) and precipitation (MAP) in the multi-source feature space allow the model to account for residual moisture effects at the statistical level; the SHAP analysis (Figure 7E) shows that SIWSI and MAP jointly contribute approximately 23% to the full model. Third, without synchronous in-situ soil moisture and surface roughness measurements, a complete physical separation of the roughness and dielectric contributions cannot be achieved; this limitation, common in regional-scale SAR-based salinity mapping [25,70,71,72,73], is discussed in Section 5.4, and future work coupling SAR retrieval with soil moisture observations (e.g., SMAP or AMSR-E) is proposed.

### 5.3. Scale Mismatch Between Satellite-Derived Signals and Ground-Based Measurements

The validity of relating satellite-derived signals to ground-based salinity measurements across varying surface conditions warrants clarification at the conceptual level. Our sampling targeted the 0–5 cm surface layer because it is the layer most directly affected by evaporative salt accumulation and the layer most directly sensed by optical and SAR sensors. Laboratory measurements on sieved (<2 mm), homogenized soil represent the bulk fine-earth fraction, whereas a 10-m satellite pixel integrates a mixture of surface elements including salt crusts, loose soil, and sparse vegetation; the model therefore retrieves the pixel-scale average surface salinity [7,74] rather than the property of any single surface element. This interpretation is consistent with field evidence from Xinjiang, where the salt content of the bare inter-row strips in mulched drip-irrigated cotton fields averaged 31.5% higher than that under the mulch [75], indicating that strong sub-pixel salinity contrasts are inherent to the dominant local cropping system.

Salt crusts increase surface reflectance—particularly in the short-wave infrared—and modify C-band backscatter through the roughness and dielectric mechanisms described in Section 5.2. Both sensing modalities consequently co-vary with salinity level at the pixel scale, even though the absolute spectral or backscatter values of a pure salt crust may differ from those of homogenized soil. Deviations can nevertheless occur under strongly crusted or freshly tilled surfaces, where the pixel-scale average may not adequately represent the bulk soil property; this limitation is discussed in Section 5.4.

### 5.4. Spatial Patterns and Prediction Uncertainty

Soil salinization in arid inland basins is closely associated with hydro-topographic gradients, with salts accumulating in low-lying alluvial plains where shallow groundwater drives capillary rise [7,76]. However, regional-scale assessments often overlook how algorithmic characteristics interact with landscape heterogeneity to produce spatially uneven prediction uncertainty, particularly in agroecotones where vegetation masks soil signals [77]. Here, we combined two machine learning models—RF and XGBoost—to map soil EC across the Tarim River Basin and scrutinized the spatial patterns and uncertainty sources using both model outputs and field measurements (Figure 9; Table 2).

Both models captured the primary longitudinal gradient of salinity: higher EC in the middle–lower reaches (alluvial plains, ancient river channels, and depressions) and lower EC in the upper mountainous areas and oasis margins (Figure 9). In the southern basin (~40° N), salinity exhibited a distinct latitudinal zonation aligned with tributary flow directions, whereas the northern mountain–plain transition zone displayed patchy salinization without clear directional organization. The RF-predicted map, when overlaid with geographical features (Figure 9), further reveals that high-salinity cores (>50 dS m^−1^) are concentrated along the middle–lower Tarim River mainstream, precisely in areas underlain by shallow groundwater (<2 m depth) and historically cultivated alluvial plains. This spatial correspondence corroborates the well-documented “concentration-dominated” regime imposed by the hyper-arid climate (precipitation 50–100 mm vs. evapotranspiration 2000–3000 mm), where negligible leaching allows salts to accumulate in flat terrains (elevation 800–1500 m, slope < 2‰) [76].

Despite comparable spatial configurations, RF and XGBoost exhibited divergent tendencies in reproducing value extremes. RF, leveraging bootstrap aggregation that tends to preserve variance, generally identified high-salinity zones more effectively, with predictions approximating the measured maximum (95.01 vs. 99.43 dS m^−1^), whereas XGBoost’s regularized gradient boosting apparently compressed the prediction range by ~23% (maximum 76.60 dS m^−1^). These observations suggest that RF may demonstrate better performance in detecting severe salinization hotspots within agriculturally modified landscapes characterized by high spatiotemporal heterogeneity (CV = 113.04%), potentially reflecting anthropogenic amplification through intensified irrigation and undrained water tables since the 1960s [76,78,79]. In particular, long-term flood irrigation without matching drainage, large-scale wasteland reclamation, and the discharge of saline drainage water into closed depressions have intensified secondary salinization in the irrigation districts of the basin. However, prediction uncertainty remained evident in both models, particularly in oasis–desert ecotones (Figure 10) where stationarity assumptions might be violated [80,81], and in vegetated areas where canopy masking introduces systematic bias [77].

This comparative assessment offers a potential framework for understanding how algorithmic choices interact with natural–anthropogenic heterogeneity in hyper-arid basins. The Tarim River Basin, hosting China’s largest endorheic river along the Taklimakan Desert rim, presents hydro-topographic challenges that limit conventional surveying [82]. By revealing divergent model behaviors in vegetation-masked agroecotones, this study provides preliminary insights for remote sensing strategies aimed at distinguishing natural from secondary salinization processes, potentially supporting precise land degradation monitoring. Future efforts might exploit time-series Sentinel-2 imagery during bare-soil periods to mitigate vegetation masking effects [83], though further validation across diverse agro-ecological settings would strengthen the generalizability of these results. Beyond methodological implications, the salinity maps produced in this study can support the dynamic monitoring and zoning-based management of soil salinization that have been identified as priorities for sustainable land management in Xinjiang [75]. The middle–lower reaches of the Tarim River Basin are among the priority areas identified for potential cropland development under irrigation scenarios in northwestern China, for which salinity assessment is a prerequisite for reclamation planning [84]. In summary, despite the limitations discussed above—namely, the scale mismatch between satellite pixels and ground measurements, the absence of synchronous in-situ observations, and the limited number of sampling points—this study reveals the regional-scale spatial patterns and model-specific uncertainty behaviours of soil salinity in the Tarim River Basin, and demonstrates the feasibility of synergistic multi-source remote sensing and machine learning for salinity retrieval in hyper-arid environments. Future work will incorporate in-situ spectral measurements of salt crusts, synchronous soil-moisture observations (e.g., from SMAP or AMSR-E), and higher-density sampling to further reduce prediction uncertainty.

## 6. Conclusions

This study has developed a reproducible framework for regional soil salinity mapping in hyper-arid environments through the fusion of multi-sensor satellite data (Sentinel-1/2), environmental covariates, and explainable machine learning. The principal conclusions are:

(1) RF with multi-source fusion outperformed single-sensor models by 10–19% (R^2^ = 0.73, RMSE = 3.58 dS m^−1^). SHAP analysis indicated a hierarchical organization of feature contributions, with climatic variables making larger contributions to model predictions than surface spectral features in the full model. XGBoost excelled at oasis–desert ecotones, suggesting synergistic use: RF for regional mapping and XGBoost for boundary delineation.
(2) Mean annual temperature and precipitation collectively contributed 33.2% to model predictions, suggesting that, in terms of relative feature contribution, soil salinity distributions in the Tarim River Basin are statistically associated with the hyper-arid climatic regime. This highlights the importance of climate-adaptive management strategies that account for negligible natural leaching capacity.
(3) Soil salinity was spatially uneven (CV = 113.0%), with higher values recorded in the middle–lower reaches relative to the upper basin, and high-salinity areas were largely coincident with zones of human activity. The generated high-resolution salinity maps provide a foundation for tiered management and future land improvement in the Tarim River Basin, and should be regarded as screening-level products that guide targeted field verification rather than plot-scale prescriptions.

## Figures and Tables

**Table 1:** Statistical summary of the EC values of the soil samples.

Soil Property	Maximum	Minimum	Mean	Median	SD	CV (%)
EC (dS m^−1^)	99.43	0.24	19.60	11.06	22.15	113.04

Note: EC (electrical conductivity), SD (standard deviation), CV (coefficient of variation).

**Table 2:** Performance comparison of machine learning models under five feature combination scenarios.

Model		SAL			VEG			SAL & VEG			SAL & VEG & POL			ALL		
		R^2^	RMSE	MAE	R^2^	RMSE	MAE	R^2^	RMSE	MAE	R^2^	RMSE	MAE	R^2^	RMSE	MAE
Traditional	PLSR	0.37	5.41	4.29	0.27	5.52	3.86	0.49	4.83	3.36	0.45	5.03	3.59	0.18	6.28	4.11
	Lasso	0.37	5.40	4.29	0.26	5.54	3.95	0.48	4.88	3.45	0.44	5.03	3.59	0.11	6.52	4.30
	GPR	0.62	3.99	2.05	0.49	4.63	2.85	0.58	3.89	2.34	0.60	4.23	1.88	0.60	3.93	2.24
	GAM	0.53	4.66	2.36	0.60	4.09	2.61	0.55	4.55	2.31	0.60	4.23	2.25	0.52	4.79	2.04
	DT	0.33	5.54	3.65	0.03	6.36	4.49	0.25	5.88	4.50	0.61	4.19	2.30	0.36	5.57	3.71
	SVM	0.29	5.74	4.64	0.26	5.54	3.98	0.48	4.88	3.61	0.44	5.02	3.48	0.22	6.10	4.16
Ensemble	LSBoost	0.60	4.31	2.29	0.56	4.28	2.53	0.46	4.97	2.73	0.71	3.63	2.03	0.68	3.89	2.10
	RF	0.60	4.30	2.96	0.62	3.98	2.53	0.67	3.89	2.33	0.73	3.48	2.10	0.73	3.58	1.89
	LightGBM	0.35	5.50	3.09	0.50	4.57	2.49	0.52	4.65	2.33	0.46	4.93	2.79	0.63	4.25	2.20
	XGBoost	0.49	4.84	3.70	0.52	4.45	3.17	0.64	3.63	2.03	0.73	3.52	1.68	0.66	4.07	1.82

Note: Unit of RMSE and MAE: dS m^−1^.

**Table 3:** Repeatability of RF and XGBoost (ALL scenario) and bootstrap 95% confidence intervals of the accuracy metrics.

Model	R^2^ (Mean ± SD)	R^2^ 95% CI	RMSE (Mean ± SD)	RMSE 95% CI	MAE (Mean ± SD)	MAE 95% CI
RF	0.70 ± 0.09	0.614–0.782	3.81 ± 0.15	3.698–3.925	1.62 ± 0.12	1.538–1.712
XGBoost	0.66 ± 0.10	0.568–0.741	3.89 ± 0.16	3.775–4.012	1.68 ± 0.13	1.592–1.778

Note: values are mean ± SD across the 100 splits; CI = percentile bootstrap 95% confidence interval from 1000 resamples of the test-set predictions within each split (tabulated CIs are the averages of the 100 split-level intervals); BCa = bias-corrected and accelerated interval. BCa 95% CIs: RF R^2^ 0.608–0.779, RMSE 3.705–3.932, MAE 1.545–1.718; XGBoost R^2^ 0.562–0.738, RMSE 3.782–4.018, MAE 1.598–1.784. Kendall’s τ of feature-importance rankings across the 100 splits: RF 0.78, XGBoost 0.72 (*p* < 0.001).

## Data Availability

The satellite-derived RS data and weather information utilized in this research are sourced from open-access repositories. Specific datasets processed and analyzed during the current study can be provided by the corresponding author upon request.

## Supplementary Materials

The following supporting information can be downloaded at: https://www.mdpi.com/article/10.3390/s26216851/s1, Supplementary information S: supporting text; Supplementary Table S1: SAR polarization parameters; Supplementary Table S2: spectral indices; Supplementary Table S3: topographic indices; Supplementary Table S4: detailed information and electrical conductivity (EC) measurements of the 147 sampling sites [85,86,87,88,89,90,91,92,93,94,95,96,97,98,99,100,101,102,103,104,105,106,107,108,109,110,111,112,113,114,115].
