# Supplementary material for: Synergistic Multi-Source Remote Sensing and Machine Learning for Regional-Scale Soil Salinity Retrieval in the Hyper-Arid Tarim River Basin
Source: Sensors (Basel). 2026 Oct 29;26(21):6851. doi: 10.3390/s26216851 (PMC13766489; doi:10.3390/s26216851)
Supplement: Supplementary file 1 [file sensors-26-06851-s001.zip › sensors-4578427-supplementary.pdf]

Supplementary Information: Materials and Methods

S1. Data Collection

S1.1 Soil Sampling and Laboratory Analysis

Soil samples were transported to the laboratory for subsequent chemical analyses. All samples were air-dried, gently crushed, homogenised, and passed through a ≤ 2 mm sieve. To prepare soil extracts, a 5:1 water-to-soil suspension was prepared by weighing 15 g of soil into a clean container and adding 75 mL of deionised water at room temperature (25 °C). After 2 h of equilibration, the electrical conductivity of the 1:5 extract ($EC_{1:5}$) was measured in triplicate at 25 °C using a calibrated conductivity meter; the mean of the three readings was taken as the representative EC value for each sampling site. $EC_{1:5}$ was employed as an index of soil salinity, following the United States Department of Agriculture (USDA) guidelines, which recognise EC as a reliable indicator of soil salinity [1].

S1.2 Satellite data acquisition and processing

Sentinel-1 synthetic-aperture radar (SAR) and Sentinel-2 optical imagery data were selected as satellite data sources. Previous studies have demonstrated that the joint use of these two sensors achieves superior accuracy for soil salinization mapping in extremely arid environments such as the Tarim River Basin [2]. All scene data were obtained free of charge from the Copernicus Open Access Hub of the European Space Agency (ESA), including: Sentinel-1 dual-polarization (VV/VH) Level-1 Ground Range Detected (GRD) images (https://dataspace.copernicus.eu, accessed on 31 May 2025) and Sentinel-2 Level-2A surface reflectance products (https://browser.dataspace.copernicus.eu, accessed on 31 May 2025).

We processed the Sentinel-1 data in SNAP 8.0 using the following chain: (1) precise orbit correction using restituted ephemerides; (2) thermal noise removal; (3) radiometric calibration to sigma-nought (σ^0); (4) speckle filtering using a Refined Lee filter with a 7 × 7 window; (5) terrain correction (Range-Doppler RTC) using the SRTM 30-m DEM; and (6) conversion of the VV and VH backscatter coefficients to decibel (dB) scale. The data comprise Level-1 Ground Range Detected (GRD) images acquired in Interferometric Wide (IW) swath mode with dual polarization (VV/VH) on an ascending orbit at an incidence angle of approximately 33 °–43 °. All scenes were acquired during the bare-soil periods of September–November 2024 and April–May 2025 to minimize vegetation cover and soil moisture confounding effects. The processed σ^0 values were resampled to 10-m resolution, co-registered with Sentinel-2 imagery using nearest-neighbor interpolation, and mosaicked and clipped to the study-area extent. A proper combination of the polarization channels of the radar images can effectively improve the correlation between the backscatter coefficient and soil salinity [3,4]. We used different polarization combinations to achieve the retrieval goal, as shown in Table S1.

We downloaded Sentinel-2 Level-2A data, which had been pre-processed with orthocorrection, geometric fine correction, and atmospheric correction using the Sen2Cor_v2.10 processor. We resampled each band to a 10-meter resolution using bilinear interpolation and merged them into multispectral images for subsequent modeling. A total of nine spectral bands were finally stacked, including Band 2 (B2, Blue), Band 3 (B3, Green), Band 4 (B4, Red), Band 5 (B5, Red Edge 1), Band 6 (B6, Red Edge 2), Band 7 (B7, Red Edge 3), Band 8 (B8, Near-Infrared), Band 11 (B11, Short-Wave Infrared 1), and Band 12 (B12, Short-Wave Infrared 2). To acquire a suitable image range for the study area, adjacent tiles were seamlessly mosaicked and clipped to the study region. Reflectance-derived spectral salinity indices and vegetation indices perform better in soil salinity inversion under vegetated

cover; hence this study employed 26 salinity indices and 28 vegetation indices (Table S2) for model development [5].

S1.3 Environmental variables (Climate and Topography)

Mean Annual Temperature (MAT) and Mean Annual Precipitation (MAP) were obtained from 13 meteorological stations of the China Meteorological Administration (CMA) within the Tarim River Basin, curated by the National Meteorological Information Center (https://data.cma.cn); the CMA network is a WMO-standardized national monitoring network. Data covered the period 1963–2023, and stations with >95% data completeness and consistent temporal coverage were prioritised. MAT and MAP surfaces (30-m resolution) were generated using inverse distance weighting (IDW) in ArcGIS 10.8 (ESRI, Redlands, CA, USA). Potential evapotranspiration (PET, 2000–3000 mm yr^{-1}) was estimated using the FAO-56 Penman–Monteith method based on the same station data. Long-term climate normals capture the aridification trends critical for soil salinity dynamics [6].

Topography exerts a critical influence on the spatial distribution of soil salinity [7]. Salinity levels are typically elevated in low-lying areas, whereas they decrease markedly in inland regions with pronounced relief and decline progressively with increasing elevation [3,8]. Consequently, topographic variables constitute key predictors in soil salinity modelling [5]. In this study, a 30-m-resolution digital elevation model (DEM) obtained from the Geospatial Data Cloud (http://www.gscloud.cn, accessed on 28 May 2025) was employed to derive 14 topographic indices using the System for Automated Geoscientific Analyses (SAGA GIS, version 7.4.0); these indices are listed in Table S3.

The candidate feature set comprised 83 parameters in total: 26 salinity indices, 28 vegetation indices, 16 environmental covariates (14 topographic indices plus MAT and MAP), and 13 SAR polarization combinations (VV, VH, and 11 derived indices; Tables S1–S3). This composition reconciles the category totals with the 83 candidate parameters subjected to Pearson correlation screening in Section S2.1.

S2. Feature Selection

A three-stage progressive feature selection framework integrating "Filter-Wrapper-Explanation" approaches was constructed, with each stage serving distinct functions: (1) Pearson correlation pre-screening (filter method) for rapid dimensionality reduction and computational efficiency; (2) Recursive Feature Elimination (RFE) (wrapper method) for optimizing feature combinations and improving prediction accuracy; and (3) SHAP (SHapley Additive exPlanations) interpretability ranking (explanation method) for revealing physical mechanisms and enhancing scientific credibility.

S2.1 Pearson-correlation Pre-screening

In machine learning-based modeling, the selection of predictive features is crucial for the accuracy of estimation outcomes [9]. Although many factors related to soil salinity can be derived through band arithmetic, these variables often exhibit inherent interdependencies. Without proper feature selection, such redundancy can not only increase model complexity but also reduce predictive performance [10]. Therefore, we employed Pearson correlation analysis to identify parameters significantly correlated with EC for subsequent salinity monitoring and mapping, thereby providing a set of potential predictors for modeling.

Using field-measured soil electrical conductivity (EC, dS m^{-1}) in the upper Tarim River drylands as the response variable, we computed the Pearson correlation coefficient (r) between EC and each of the 83 candidate features (13 polarimetric indices, 26 salinity indices, 28 vegetation indices and 16 environmental covariates). The larger $|r|$, the stronger the linear salinity association [5]. Variables that passed the 0.05 significance test

were selected to participate in the modeling, reducing dimensionality from 83 to 49 and guaranteeing that all selected features are linearly related to soil EC.

S2.2 RFE Recursive Refinement

Recursive feature elimination (RFE) with an embedded random forest (RF) evaluator was subsequently applied [9]. RF models were trained under 10-fold cross-validation; features were ranked by Gini importance and the least-important variable was discarded in each iteration. The subset yielding the minimum root-mean-square error (RMSE) was chosen, resulting in a final parsimonious set of 42 polarimetric-spectral-environmental covariates that optimally balance predictive accuracy and redundancy.

S2.3 Interpretable Ranking of Refined Features

To evaluate the applicability and robustness of various data and feature sets in soil salinity modeling, we divided the 42 refined features into five thematic scenarios: (i) salinity indices only (SAL), (ii) vegetation indices only (VEG), (iii) salinity & vegetation indices (SAL & VEG), (iv) salinity & vegetation & polarimetry (SAL & VEG & POL), and (v) all features (ALL). To enhance model accuracy and efficiency, we employed the eXtreme Gradient Boosting (XGBoost) algorithm and SHAP (Shapley Additive Explanations) values to identify the most relevant features for each scenario [11−12]. XGBoost, a gradient boosting algorithm, is highly effective in predictive modeling, particularly with large and complex datasets. SHAP values were used to quantify the contribution of each feature, providing an interpretable measure of feature importance within the model [12]. Across all scenarios, hyperparameters were fixed: boosting_type = gbdt, learning_rate = 0.1, n_estimators = 100. After 10-fold cross-validation training, the mean absolute SHAP value of each feature was computed and visualised as a stacked bar chart. Feature selection techniques are critical in this process to optimize model input and mitigate overfitting [8,13]. While most research focuses on model predictive performance, there is a growing emphasis on model interpretability using methods like SHAP to uncover the driving factors behind predictions [11].

S3. Modeling framework

We employed ten machine learning algorithms to model soil electrical conductivity (EC) under each scenario: Traditional models (Partial Least Squares Regression (PLSR), Least Absolute Shrinkage and Selection Operator (LASSO), Gaussian Process Regression (GPR), Generalized Additive Model (GAM), Decision Tree (DT) and Support Vector Machine (SVM)), and Ensemble methods (Least-Squares Boosting (LSBoost), Random Forest (RF), Light Gradient Boosting Machine (LightGBM) and eXtreme Gradient Boosting (XGBoost)). In total, this resulted in 50 model runs (5 scenarios × 10 algorithms).

The dataset was partitioned into training (80%, n = 118) and independent testing (20%, n = 29) subsets. Within the training set, 10-fold cross-validation combined with Bayesian hyperparameter optimization was employed for model selection and hyperparameter tuning. The optimal model was subsequently evaluated on the held-out testing set. Evaluation metrics included the coefficient of determination (R^2), root mean square error (RMSE), and mean absolute error (MAE). The analysis was conducted using MATLAB R2025a (MathWorks, Natick, MA, USA). The best-performing machine learning algorithm with optimized hyperparameters was selected and subsequently applied to generate a spatial map of soil electrical conductivity (EC) across the entire study area.

S4. Soil EC mapping

Based on the model evaluation results (Section S3), the optimal model (RF) and the suboptimal model (XGBoost) were selected for spatial extrapolation to generate soil EC

distribution maps for the entire study area. The prediction process involved applying the trained models to raster stacks of the selected features (spectral indices, SAR parameters, and environmental covariates) to produce continuous soil salinity surfaces at the corresponding spatial resolution.

S5. Supplementary Tables

All polarization combinations were calculated from σ^0 in linear power units before conversion to dB for visualization. Explicit definitions (in the order of Table S1): (1) VV + VH: sum of co-polarized and cross-polarized backscatter, sensitive to total scattering intensity; (2) VV − VH: difference between co- and cross-polarized backscatter, related to polarization ratio and surface roughness; (3) $VV^2 + VH^2$: sum of squared backscatter coefficients, proxy for total scattered energy; (4) $VV^2 - VH^2$: difference in squared coefficients, related to dielectric contrast; (5) $VV^2 + VH$: sum of squared co-polarized backscatter and cross-polarized backscatter, an asymmetric power combination of the two channels; (6) $VH^2 - VV$: difference between squared cross-polarized and co-polarized backscatter, an asymmetric power combination; (7) $VV^2 - VH$: difference between squared co-polarized and cross-polarized backscatter, an asymmetric power combination; (8) $(VV^2 + VH^2)/VH$ and (9) $(VV^2 + VH^2)/VV$: ratio-based indices sensitive to surface scattering mechanisms (roughness-dominated vs. dielectric-dominated); (10) $(VV^2 - VH^2)/VV$: normalized difference index, highlighting relative polarization differences; (11) $(VV^2 + VH^2)/(VV^2 - VH^2)$: contrast index, emphasizing scattering anisotropy.

Table S1. Polarization combinations used in the study.

Polarization mode combination	Reference	Polarization mode combination	Reference
VV	[3]	VH^2-VV	[3]
VH	[3]	VV^2-VH	[3]
VV+VH	[3]	$(VV^2+VH^2)/VH$	[4]
VV-VH	[3]	$(VV^2+VH^2)/VV$	[4,14]
VV^2+VH^2	[3]	$(VV^2-VH^2)/VV$	[4]
VV^2-VH^2	[4]	$(VV^2+VH^2)/(VV^2-VH^2)$	[4]
VV^2+VH	[3]		

Note: VV and VH denote the backscatter coefficients (σ^0) of the co-polarized and cross-polarized channels, respectively; the combinations were calculated in linear power units and are expressed in decibels (dB).

Table S2. Spectral Indices used in the study.

Category	Acronym	Formula	Reference	Acronym	Formula	Reference
Salinity Indices	B2	B2	[15,16]	S7	(B3+B4)/2	[17]
	B3	B3	[15,16]	S8	(B3+B4+B8)/2	[17]
	B4	B4	[15,16]	SI-T	B4/B8×100	[17]
	B5	B5	[15,16]	SI	$(B2×B4)^{1/2}$	[17]
	B8	B8	[15,16]	SI1	$(B3^2+B4^2+B8^2)^{1/2}$	[18]
	B11	B11	[15,16]	SI2	$(B3^2+B4^2)^{1/2}$	[19]
	B12	B12	[15,16]	SI3	$(B3×B4)^{1/2}$	[17]
	S1	B2/B4	[20]	SI4	B11/B8	[17]
	S2	(B2-B4)/(B2+B4)	[17,20]	NDSI	(B4-B8)/(B4+B8)	[19]
	S3	B3×B4/B2	[20]	SSSI1	B11-B12	[21]
	S4	(B2×B4)/B3	[17]	SSSI2	(B11×B12-B12×B12)/B11	[21]
	S5	(B4×B8)/B3	[20]	SAIO	(B4-B8)/(B3+B8)	[22]
	S6	(B11-B12)/(B11+B12)	[21,23]	CRSI	$((B8×B4-B3×B2)/B5×B4+B3×B2)^{1/2}$	[23]
Vegetation Indices	B2	B2	[15,16]	GNDVI	(B8-B3)/(B8+B3)	[24]
	B3	B3	[15,16]	DVI	B8-B4	[25]
	B4	B4	[15,16]	NDGI	(B3-B4)/(B3+B4)	[21]
	B5	B5	[15,16]	ENDVI	(B8+B12-B4)/(B8+B12+B4)	[21]
	B8	B8	[15,16]	COSRI	(B2+B3)/(B4+B8)×(B4-B8)/(B4+B8)	[32]
	B11	B11	[15,16]	MSAVI	$0.5×(2×B8+1-((2×B8+1)^2-8×(B8-B4))^{1/2})$	[27]
	B12	B12	[15,16]	NDWI	(B3-B8)/(B3+B8)	[28]
	NDVI	(B8-B4)/(B8+B4)	[29]	CiGREEN	B8/B3	[21]
	DFI	$(1-\frac{B12}{B11})×\frac{B4}{B8}×100$	[30]	RVI	B8/B4	[21]
	SAVI	(B8-B4)/(B8+B4+0.5)×1.5	[31]	NDI	(B12-B7)/(B12+B7)	[21]
	OSAVI	(B8-B4)/(B8+B4+0.16)	[32]	ENDVI1	（B8+B11-B4/(B8+B12+B4)	[33]
	EVI	2.5×(B8-B4)/(1+B8+6×B4-7.5×B2)	[34]	GVMI	[(B8+0.1)-(B11+0.02)]/[(B8+0.1)+(B11+0.02)]	[21]
	EEVI	2.5×(B8-B11-B4)/(B8+B11+6×B4-7.5×B2+1)	[35]	ARVI	(B8-(2×B4-B2))/(B8+(2×B4-B2))	[36]
	RDVI	$(B8-B4)/(B8+B4)^{1/2}$	[37]	SIWSI	(B12-B8)/(B12+B8)	[21]

Table S3. Topography indices and acronyms used in this study.

Topography index	Index acronym	Reference
Digital Elevation Model(m)	DEM	[38]
Slope	S	[38]
Aspect	AS	[38]
Analytical Hillshading	AH	[38]
Convergence Index	CI	[38]
Plan Curvature	SC	[38]
Profile Curvature	PC	[38]
Total Catchment Area	TCA	[38]
Ls Factor	LSF	[38]
Channel network base level	CNBL	[38]
Channel network distance	CND	[38]
Valley depth	VD	[38]
Relative slope position	RSP	[38]
Topographic Wetness Index	TWI	[38]

Table S4. Detailed information and electrical conductivity (EC) measurements of the 147 sampling sites.

Type	Sample ID	EC(dS m^{-1})	Longitude (°E)	Latitude (°N)	Notes
cropland	TMG-1-2	5.6513	85.7911	41.8142	Cotton field soil sample
	TMG-1-12	1.7189	85.6369	41.7888	Cotton field soil sample
	ALR-1	2.941	81.216	40.432	Winter wheat field
	ALR-13	0.2431	79.936	40.477	Cotton field
	ALR-17	3.8419	79.758	40.551	Sunflower field
	ALR-19	1.3223	79.753	40.551	Cropland
	ALR-23	27.3943	79.951	40.621	Cropland
	ALR-25	0.8997	80.48	40.643	Cotton field
	ALR-36	4.2443	80.642	40.621	Cropland on left side of salt drainage ditch
	ALR-38	11.0644	80.714	40.608	Reclaimed cropland
	ALR-40	12.4246	80.713	40.608	Cropland soil profile
	TMSK-7	10.5252	80.3567	40.2987	Wheat field
	TMSK-12	35.4211	80.329	40.2806	Cotton field
	AKS-3	2.4245	81.154	40.717	Wheat field
	AKS-8	1.0781	81.069	40.861	Cotton field
	AKS-16	3.3177	80.816	41.296	Cotton field
	AKS-18	1.9063	80.814	41.362	Wheat field
	AKS-32	0.6326	80.821	41.37	Apple orchard
	AKS-33	1.854	80.801	41.336	Cotton field
	AKS-34	1.8968	80.813	41.282	Cotton field
	AKS-36	3.4927	80.802	41.271	Wheat field
	SY-4	0.6889	81.424	40.482	Cotton field soil sample
	SY-6	2.606	81.473	40.493	Cropland
	SY-8	0.8213	81.494	40.51	Wheat field
	SY-31	2.0152	81.462	40.706	Cotton field soil sample
	TMSK-2-2	0.7433	79.3725	40.047	Cropland soil sample
	TMSK-2-3	1.6732	79.968	40.0379	Cotton field
	TMSK-2-8	1.0967	79.137	39.8728	Cotton field
	TMSK-2-9	0.3337	79.137	39.8728	Plastic film contamination
	TMSK-2-15	7.007	79.211	39.8687	Wheat field soil sample
	TMSK-2-16	2.569	79.211	39.8687	Wheat field soil profile
	TMSK-2-18	1.7742	79.2229	39.8675	Cropland soil sample
	TMSK-2-28	0.5028	79.0752	39.8251	Maize field soil sample
	TMSK-2-33	2.5685	79.013	39.9642	Newly reclaimed land
	TMSK-2-34	2.7708	79.013	39.9642	Cotton field

	TMSK-2-35	9.237	79.0065	39.9587	Maize field soil profile
	TMSK-2-67	0.9949	79.4503	39.9658	Cropland soil sample
	TMSK-2-70	0.5488	79.5353	39.888	Cropland soil sample
	TMSK-2-72	0.6806	79.5473	39.8808	Wheat field soil sample
	SY-2-7	0.2706	82.7027	41.1888	Wheat field
	SY-2-9	1.9577	82.8421	41.3695	Wheat field
	SY-2-11	3.8556	82.9519	41.3936	Cotton field soil sample
	ALR-6	36.7854	80.755	40.357	Surface soil sample from unused saline-alkali land
	ALR-7	8.7285	80.755	40.357	Borehole sample
	ALR-10	79.3738	80.619	40.355	Unused saline-alkali land
	ALR-11	58.712	80.512	40.318	Unused saline-alkali land
	ALR-12	17.1312	80.512	40.318	Saline-alkali soil profile
	ALR-14	44.2633	79.936	40.477	Unused saline-alkali land
	ALR-15	29.9006	79.76	40.55	Saline-alkali land
	ALR-21	20.7984	79.963	40.567	Unused saline-alkali land
	ALR-24	3.8967	80.48	40.643	Unused saline-alkali land
	ALR-29	11.4095	80.527	40.638	Reclaimed saline-alkali land with fruit seedlings
	ALR-30	64.4799	80.537	40.638	Reed saline-alkali land
	ALR-32	13.6657	80.586	40.583	Soil sample from drainage ditch
	ALR-34	12.816	80.642	40.62	Unused saline-alkali land (right side of salt discharge channel)
	TMSK-3	20.6888	80.3706	40.4673	Unused saline-alkali land
	TMSK-11	41.3213	80.329	40.2806	Tamarix saline-alkali land
	TMSK-16	84.1793	80.528	40.3232	Unused saline-alkali land
	AKS-7	2.3347	81.069	40.861	Unused reclaimed land
	AKS-12	65.4067	80.976	41.014	Unused saline-alkali land
saline-alkali land	AKS-13	54.1867	80.917	41.112	Unused saline-alkali land
	AKS-21	99.4288	80.765	41.557	Gypsum from saline hill
	AKS-22	36.503	80.765	41.557	Gypsum from saline hill
	AKS-23	20.9235	80.765	41.557	Red saline stone (soil)
	SY-16	52.305	81.754	40.586	Unimproved land
	SY-18	2.0547	81.754	40.586	Land under improvement
	SY-19	26.9521	81.727	40.58	Saline-alkali wasteland
	SY-20	43.7768	81.754	40.603	Unused saline-alkali land
	SY-21	39.1641	81.878	40.645	Unused saline-alkali land
	SY-27	6.3345	81.487	40.693	Ameliorated saline-alkali land
	SY-32	68.8658	81.462	40.706	Unused saline-alkali land
	TMSK-2-5	24.9694	79.2894	40.0163	Unused saline-alkali land
	TMSK-2-13	37.5629	79.2145	39.8696	Unused saline-alkali land
	TMSK-2-14	15.6044	79.2145	39.8696	Profile
	TMSK-2-19	6.2782	79.2435	39.851	Unused saline-alkali land
	TMSK-2-38	4.6129	78.9942	39.966	Desalination pit
	TMSK-2-39	26.3546	78.9879	39.963	Unused saline-alkali land
	TMSK-2-40	10.9793	78.975	39.9543	Unused saline-alkali land
	TMSK-2-41	27.7117	78.9693	39.9654	Unused saline-alkali land
	TMSK-2-44	18.0126	78.9618	39.9397	Unused saline-alkali land
	TMSK-2-45	27.205	78.979	39.9353	Unused saline-alkali land
	TMSK-2-55	14.9162	78.9098	39.8147	Unused saline-alkali land
	TMSK-2-66	4.6107	79.4503	39.9658	Unused saline-alkali land
	TMSK-2-68	0.3552	79.4672	39.9032	Unused saline-alkali land
	KC-1-3	11.5362	83.4875	41.5025	Unused land
	LT-1-14	29.3173	83.5407	41.1384	Wasteland
	LT-1-16	43.9607	83.4429	41.0942	Wasteland
grassland & unused land	KC-1-4	38.6905	83.7798	41.5025	Unused land
	TMG-1-6	28.9978	85.5905	41.8611	Unused land
	TMG-1-8	33.9307	85.5797	41.8695	Unused land
	TMG-1-14	44.2188	85.6228	41.7759	Soil sample from unused land

	TMG-1-15	25.6811	85.6178	41.5799	Unused land
	TMG-1-17	55.856	85.4433	41.8509	Unused land
	TMG-1-18	58.9817	85.4041	41.8398	Unused land
	TMG-1-20	53.7154	85.3613	41.8784	Unused land
	TMG-1-21	47.5037	85.3946	41.8524	Unused land
	TMG-1-22-0	11.1548	85.3975	41.8474	Unused land
	TMG-1-23	7.46	85.1211	41.7591	Unused land
	TMG-1-25	31.5545	84.7961	41.6146	Unused land
	TMG-1-26	2.71	84.5879	41.518	Unused land
	TMG-1-27	16.7639	84.44	41.4837	Unused land
	TMG-1-28	2.9534	84.3019	41.5884	Unused land
	TMG-1-29	5.5123	84.3167	41.6269	Unused land
	LT-1-1	41.7732	84.2965	41.5852	Unused land
	LT-1-2	2.34	84.1415	41.4822	Unused land
	LT-1-3	0.9961	84.0696	41.4737	Unused land with tamarisk (*Tamarix* spp.)
	LT-1-6	0.5893	83.819	41.2854	Unused land
	LT-1-10	7.8252	83.7415	41.2184	Wasteland
	LT-1-12	82.9492	83.6783	41.1823	Unused land
	ALR-28	81.5982	80.522	40.655	Abandoned reed field
	ALR-37	85.6619	80.714	40.607	Unused land
	AKS-4	45.7944	81.125	40.765	Unused land
	AKS-9	26.196	81.032	40.921	Wasteland with tamarisk (Tamarix) vegetation
	AKS-11	25.1395	81.032	40.921	Stratigraphic profile with interbedded sand and red clay
	AKS-20	0.4343	80.776	41.497	Alluvial fan plain
	AKS-27	0.629	80.777	41.753	Meadow soil
	AKS-31	1.6917	80.725	41.704	Soil sample
	SY-12	51.7544	81.548	40.541	Reed bed
	SY-25	4.3004	81.851	40.566	Reed bed
	SY-29	0.2445	81.487	40.694	Floodplain
	TMSK-2-47	3.4576	79.0105	39.9541	Soil sample from unused land
	TMSK-2-50	23.3074	78.9857	39.9092	Unused land
	TMSK-2-52	17.7873	78.9735	39.875	Unused land
	SY-2-8	18.4318	82.8421	41.3695	Wasteland
	SY-2-13	34.5942	83.0244	41.4057	Wasteland soil sample
	LT-1-21	15.9518	83.152	41.0743	Unused wasteland
forest & shrubland	LT-1-18	26.8009	83.3012	41.0033	Poplar forest
	TMG-1-4	4.5706	85.7612	41.82	Forest soil sample
	LT-1-8	23.2373	83.7694	41.2514	Populus euphratica forest land
	TMSK-8	53.0232	80.3567	40.2987	Populus euphratica forest land
	TMSK-13	3.8475	80.2658	40.2123	Populus euphratica forest land
	TMSK-14	18.9411	80.2751	40.2238	Populus euphratica forest land
	TMSK-15	2.079	80.2848	40.2404	Haloxylon forest land
	AKS-14	24.0579	80.842	41.238	Haloxylon forest land
	AKS-39	21.6798	80.89	41.157	Populus euphratica forest land
	SY-1	21.9005	81.369	40.48	Populus euphratica forest land
	SY-23	0.8428	82.04	40.728	Populus euphratica forest land
	TMSK-2-11	0.6986	79.2119	39.8734	Forest soil sample
	TMSK-2-22	10.5342	79.237	39.7961	Populus euphratica forest land
	TMSK-2-61	7.4581	79.272	39.9398	Populus euphratica forest land

TMSK-2-63	6.727	79.3729	39.9177	Populus euphratica forest land
TMSK-2-65	2.7119	79.4258	39.9137	Populus euphratica forest land
TMSK-2-73	14.1468	79.5484	39.8648	Populus euphratica forest land
TMSK-2-75	10.5008	79.5628	39.8369	Populus euphratica forest land
SY-2-2	14.6595	82.1458	40.8702	Shrubland
SY-2-6	21.8337	82.3201	41.0419	Shrubland
LT-1-22	2.1641	82.1089	40.8156	Haloxylon forest land

References

1. Schoeneberger, P.J.; Wysocki, D.A.; Benham, E.C.; Soil Survey Staff. *Field Book for Describing and Sampling Soils*, Version 3.0; Natural Resources Conservation Service, National Soil Survey Center: Lincoln, NE, USA, **2012**; Available online: https://www.nrcs.usda.gov/resources/guides-and-instructions/field-book-for-describing-and-sampling-soils (accessed on 15 October 2024).
2. Ma, C. Quantitative retrieval of soil salt content based on Sentinel-1 dual polarization radar image. *Trans. Chin. Soc. Agric. Eng*. **2018**, 34(2), 153–158. 10.11975/j.issn.1002-6819.2018.02.021. (In Chinese with English abstract)
3. Ma, G.; Ding, J.; Han, L.; Zhang, Z.; Ran, S. Digital mapping of soil salinization based on Sentinel-1 and Sentinel-2 data combined with machine learning algorithms. *Reg. Sustain*. **2021**, 2, 177–188. 10.1016/j.regsus.2021.06.001.
4. Zhang, Q.; Zhou, Z.S.; Caccetta, P.; Simons, J.; Li, L. Sentinel-1 imagery incorporating machine learning for dryland salinity monitoring: A case study in Esperance, Western Australia. *Int. J. Appl. Earth Obs. Geoinf.* **2022**, *112.* 10.1109/IGARSS39084.2020.9323426.
5. Wang, Z.; Zhang, F.; Zhang, X.; Chan, N.W.; Kung, H.T.; Ariken, M.; Zhou, X.; Wang, Y. Regional suitability prediction of soil salinization based on remote-sensing derivatives and optimal spectral index. *Sci. Total Environ.* **2021**, *775.*
6. Bannari, A.; Al-Ali, Z. Assessing climate change impact on soil salinity dynamics between 1987–2017 in arid landscape using Landsat TM, ETM+ and OLI data. *Remote Sens.* **2020**, *12(17), 2794.* 10.3390/rs12172794
7. Gallant, J.C.; Dowling, T.I. A multiresolution index of valley bottom flatness for mapping depositional areas. *Water Resour. Res.* **2003**, *39(12).* 10.1029/2002WR001426
8. Li, J.; Zhang, T.; Shao, Y.; Ju, Z. Comparing machine learning algorithms for soil salinity mapping using topographic factors and Sentinel-1/2 data: A case study in the Yellow River Delta of China. *Remote Sens.* **2021**, *15(9), 2332.* 10.3390/rs15092332
9. Fathizad, H.; Hakimzadeh Ardakani, M.A.; Sodaiezadeh, H.; Kerry, R.; Taghizadeh-Mehrjardi, R. Investigation of the spatial and temporal variation of soil salinity using random forests in the central desert of Iran. *Geoderma* **2020**, *365.* 10.1016/j.geoderma.2020.114233
10. Peng, J.; Biswas, A.; Jiang, Q.; Zhao, R.; Hu, J.; Shi, Z. Estimating soil salinity from remote sensing and terrain data in southern Xinjiang Province, China. *Geoderma* **2018**, *337.* 10.1016/j.geoderma.2018.08.006
11. Aksoy, E.; Sertel, E.; Roscher, R.; Tanik, A.; Hamzehpour, N. Assessment of soil salinity using explainable machine learning methods and Landsat 8 images. *Int. J. Appl. Earth Obs. Geoinf.* **2024**, *130, 103879.* 10.1016/j.jag.2024.103879
12. Kaplan, G.; Gašparović, M.; Alqasemi, A.S.; Aldhaheri, A.; Abuelgasim, A.; Ibrahim, M. Soil salinity prediction using machine learning and Sentinel-2 remote sensing data in hyper-arid areas. *Phys. Chem. Earth Parts A/B/C* **2023**, *130, 103400.* https://doi.org/10.1016/j.pce.2023.103400.
13. Wang, N.; Xue, J.; Peng, J.; Biswas, A.; He, Y.; Shi, Z. Integrating remote sensing and landscape characteristics to estimate soil salinity using machine learning methods: A case study from Southern Xinjiang, China. *Remote Sens.* **2020**, *12(24), 4118.* 10.3390/rs12244118
14. Hihi, S.; Katlane, R.; Kilani, B.; Zekri, M.W.; Bensalah, R.; Siewert, C.; Kallel, M. Evaluating drought effects on soil: Innovative soil salinity monitoring via SAR data, Sentinel-2 imagery, and machine learning algorithms in Kerkennah Archipelago. *Atmosphere* **2023**, *14(10), 1514.* 10.3390/atmos14101514
15. Drusch, M.; Del Bello, U.; Carlier, S.; Colin, O.; Fernandez, V.; Gascon, F.; Hoersch, B.; Isola, C.; Laberinti, P.; Martimort, P.; Meygret, A.; Spoto, F.; Sy, O.; Marchese, F.; Bargellini, P. Sentinel-2: ESA's optical high-resolution mission for GMES operational services. *Remote Sens. Environ.* **2012**, *120*, 25–36.

16. ESA [European Space Agency]. *Sentinel-2 User Handbook*; ESA Standard Document, Issue 1; ESA Communications: Paris, France, **2015**; Available online: https://sentinel.esa.int/documents/247904/685211/Sentinel-2_User_Handbook (accessed on 31 May 2025).
17. Douaoui, A.E.K.; Nicolas, H.; Walter, C. Detecting salinity hazards within a semiarid context by means of combining soil and remote-sensing data. *Geoderma* **2006**, *134*, 217–230. 10.1016/j.geoderma.2005.10.009
18. Khan, N.M.; Rastoskuev, V.V.; Shalina, E.V.; Sato, Y. Mapping salt-affected soils using remote sensing indicators—a simple approach with the use of GIS IDRISI. In *Proceedings of the 22nd Asian Conference on Remote Sensing*, Singapore, 9–13 November 2001.
19. Khan, N.M.; Sato, Y.; Shiozawa, S. Assessment of hydrosaline land degradation by using a simple approach of remote sensing indicators. *Agric. Water Manag.* **2005**, *77, 96–109.* https://doi.org/10.1016/j.agwat.2004.09.038.
20. Khan, S.; Abbas, A. Using remote sensing techniques for appraisal of irrigated soil salinity. In *Proceedings of the International Congress on Modelling and Simulation (MODSIM 2007)*, Christchurch, New Zealand, 10–13 December **2007**; pp. 2632–2638.
21. Bannari, A.; Guedon, A.M.; El-Harti, A.; Zaghden, H.; Teodorov, C. Characterization of slightly and moderately saline and sodic soils in irrigated agricultural land using simulated data of advanced land imaging (EO-1) sensor. *Commun. Soil Sci. Plant Anal.* **2008**, *39(19–20), 2795–2811.* 10.1080/00103620802432717.
22. Allbed, A.; Kumar, L.; Sinha, P. Mapping and modelling spatial variation of soil salinity in the Al Hassa oasis based on remote sensing indicators and regression techniques. *Remote Sens.* **2014**, *6(2), 1137–1157.* 10.3390/rs6021137.
23. Scudiero, E.; Skaggs, T.H.; Corwin, D.L. Regional-scale soil salinity assessment using Landsat ETM+ reflectance. *Remote Sens. Environ.* **2014**, *140, 335–343.* 10.1016/j.rse.2015.08.026.
24. Gitelson, A.A.; Kaufman, Y.J.; Merzlyak, M.N. Detection of red edge position and chlorophyll content by reflectance measurements near 700 nm. *J. Plant Physiol.* **1996**, *148, 501–508.* 10.1016/S0176-1617(96)80285-9.
25. Tucker, C.J. Red and photographic infrared linear combinations for monitoring vegetation. *Remote Sens. Environ.* **1979**, *8, 127–150.* https://doi.org/10.1016/0034-4257(79)90013-0.
26. Abuelgasim, A.; Ammad, R. Mapping soil salinity in arid and semi-arid regions using Landsat 8 OLI satellite data. Remote Sens. Appl. Soc. Environ. 2019, 13, 415–425. https://doi.org/10.1016/j.rsase.2018.12.010
27. Qi, J.; Chehbouni, A.; Huete, A.R.; Kerr, Y.H.; Sorooshian, S. A modified soil adjusted vegetation index. *Remote Sens. Environ.* **1994**, *48 119 -126.* 10.1016/0034-4257(94)90134-1.
28. McFeeters, S.K. The use of the Normalized Difference Water Index (NDWI) in the delineation of open water features. *Int. J. Remote Sens.* **1996**, *17, 1425–1432.* https://doi.org/10.1080/01431169608948714.
29. Rouse, J.W.; Haas, R.H.; Schell, J.A.; Deering, D.W. Monitoring vegetation systems in the Great Plains with ERTS. In *Proceedings of the Third Earth Resources Technology Satellite-1 Symposium*, Washington, DC, USA, **1974; pp. 309–317.**
30. Gao, Y.; Xue, X.; Zhang, Y.; Liao, J. Remote sensing monitoring of soil salinity in the Hetao Irrigation District. *Science Press*: *Beijing, China*, **2010**. (In Chinese).
31. Huete, A.R. A soil-adjusted vegetation index (SAVI). *Remote Sens. Environ.* **1988**, *25, 295–309.* https://doi.org/10.1016/0034-4257(88)90106-X.
32. Rondeaux, G.; Steven, M.; Baret, F. Optimization of soil-adjusted vegetation indices. *Remote Sens. Environ.* **1996**, *55, 95–107.* https://doi.org/10.1016/0034-4257(95)00186-7.
33. Ba, Y.; Zhang, Z.; Xie, P.; Fan, S.; Du, R.; Guo, F.; Qian, L.; Bai, X.; He, Y.; Fan, S. Inverting soil salinity of farmland in Xinjiang by integrating Sentinel-1/2 and environmental variables. *Trans. Chin. Soc. Agric. Eng.* **2024**, *40*, 171–179. 10.11975/j.issn.1002-6819.202401070. (In Chinese with English abstract).
34. Huete, A.; Didan, K.; Miura, T.; Rodriguez, E.P.; Gao, X.; Ferreira, L.G. Overview of the radiometric and biophysical performance of the MODIS vegetation indices. *Remote Sens. Environ.* **2002**, *83, 195–213.* 10.1016/S0034-4257(02)00096-2.
35. Chen, H.; Zhao, G.; Chen, J.; Wang, R.; Gao, M. Remote sensing inversion of saline soil salinity based on modified vegetation index in estuary area of Yellow River. *Trans. Chin. Soc. Agric. Eng.* **2015**, *31*, 107–114. https://doi.org/10.3969/j.issn.1002-6819.2015.05.016. (In Chinese with English abstract).
36. Kaufman, Y.J.; Tanré, D. Atmospherically resistant vegetation index (ARVI) for EOS-MODIS. *IEEE Trans. Geosci. Remote Sens.* **1992**, *30, 261–270.* 10.1109/36.134076.
37. Roujean, J.L.; Breon, F.M. Estimating PAR absorbed by vegetation from bidirectional reflectance measurements. *Remote Sens. Environ.* **1995**, *51, 375–384.* https://doi.org/10.1016/0034-4257(94)00114-3.
38. Conrad, O.; Bechtel, B.; Bock, M.; Dietrich, H.; Fischer, E.; Gerlitz, L.; Wehberg, J.; Wichmann, V.; Bohner, J. System for Automated Geoscientific Analyses (SAGA) v. 2.1.4. *Geosci. Model Dev.* **2015**, *8, 1991–2007.* https://doi.org/10.5194/gmd-8-1991-2015.
